# Quantitative analysis of the labia minora morphology in 400 Chinese women: A new method for assessing the shape of the labia minora

**DOI:** 10.3389/fsurg.2022.961247

**Published:** 2023-01-06

**Authors:** Kexin Che, Keke Wang, Ye Yuan, Fengyong Li, Qiang Li

**Affiliations:** Gynecological Plastic Surgery Department, Plastic Surgery Hospital, Chinese Academy of Medical Sciences and Peking Union Medical College, Beijing, China

**Keywords:** labia minora, morphology, shape features, labiaplasty, vulva

## Abstract

**Purpose:**

To investigate the shape of labia minora in Chinese adult women.

**Methods:**

Women who visited the Genital Plastic Surgery Center from January 2021 to February 2022 were included and the vulvar regions were photographed. The shape of the labia minora was converted into quantifiable values [left side line segment 1–9 (*L1–L9* values) and right side line segment 1–9 (*R1–R9* values)]. The 400 enrolled patients were grouped by age, parity, and the types of vulvar surgeries they planned to undergo.

**Results:**

After a graphic-to-digital and digital-to-graphic conversion, the simulated average image of the labia minora in Chinese adult women was constructed based on the mean values of *L1–L9* and *R1–R9*. Comparing the values of *Ln* and *Rn* in the four age groups revealed that the mean values of *Ln* and *Rn* gradually decreased with age, but only two subgroups showed statistically significant differences (*P*-value <0.05). When the patients were stratified according to the number of births and whether labiaplasty was planned to perform, there were no significant differences among all groups (*P*-value >0.05).

**Conclusion:**

To measure and evaluate the labia minora, a new assessing method was used. It is an innovative attempt to transform the simple rough description of the shape of the labia minora into more precise data reflecting the shape features. It allows the comparison of labia minora shape between individuals by comparing quantifiable values. Besides, the average shape of labia minora in Chinese adult women was provided visually. Factors including age, parity, and whether the patient planned to undergo labiaplasty were not significantly associated with the shape of the labia minora.

## Introduction

The popularity of female genital plastic surgery is gradually rising, and so is labiaplasty (1). Enlarged labia minora could cause functional, cosmetic, and psychosocial issues (2). In 1984, the first article describing labiaplasty was published and edge trim techniques were used for three patients who felt the protrusion of the labia minora was aesthetically and functionally unsatisfactory (3). Indeed, the length, width, and color of the labia minora can vary widely. The first study regarding genital dimensions in healthy women was published by Lloyd et al. in 2005 (4). When the transverse distance from the interlabial groove to the widest part of the labia minora is greater than 40–50 mm, it could be diagnosed as labia minora hypertrophy (5). Many plastic surgeons have previously measured and described the female vulva and defined the hypertrophic labia minora (5–8). Nevertheless, there is currently no standard for an aesthetically perfect labia minora (9). Culturally projected interpretation biases toward one's genital appearance could cause concerns compelling patients to seek female genital plastic surgeries (10). The general shape of the labia minora has a far-reaching impact on the patient's willingness to undergo labiaplasty and choices of surgical strategies. Female genital parameters have been surveyed in the United Kingdom, China, the United States, Denmark, India, Australia, Israel, etc. (4, 9, 11–15).

Although plastic surgeons and gynecologists have taken measurements of the labia minora, such as the labial length and labial width (16). No scholars have quantitatively analyzed the morphological characteristics of the labia minora. This study is the first morphological study of the labia minora in a large cohort. To provide a better understanding of the morphological features of the labia minora, we present a descriptive study addressing labia minora measurement. Herein, the shape of the labia minora was converted to numerical values for an in-depth comparative analysis of 400 Chinese adult women.

## Methods

### Patients

Women who visited the Genital Plastic Surgery Center with various surgical intentions were consecutively included in the study. Patients were excluded if they had a history of vulvar surgeries before they visited the Genital Plastic Surgery Center because the labia minora may no longer be in its primitive, non-interventionist state. A total of 400 female patients were included in chronological order. Data were collected on age, the number of births, and surgical information (Supplementary File 4). The included consecutive patients underwent surgeries in the vulvar region in the Genital Plastic Surgery Center between January 2021 and February 2022 and the measurements of the labia minora were all scheduled before surgery. Left side labiaplasty was performed on 21 patients, right side labiaplasty was performed on 28 patients, bilateral labiaplasty was performed on 173 patients, clitoral hood reduction was performed on 185 patients, vaginal tightening surgical procedure was performed on 122 patients, labia majora augmentation was performed on 29 patients, hymen repair was performed on 60 patients, and other procedures in the vulva area such as the excision of pigmented nevus and masses were performed on 22 patients (Supplementary File 4). Labiaplasty included 149 cases of trim labiaplasty and 73 cases of wedge labiaplasty. The vaginal tightening procedures mentioned above refer to the excision of redundant vaginal tissues, the imbrication of the median levator muscles, and the remodeling of the perineal body.

This study was approved by the appropriate institutional research ethics committee. Photographs were obtained with written informed consent, and all data were analyzed anonymously according to the principles in the Declaration of Helsinki 1975 (revised in 2008).

### Measurements and statistical analysis

**Figure 1:**
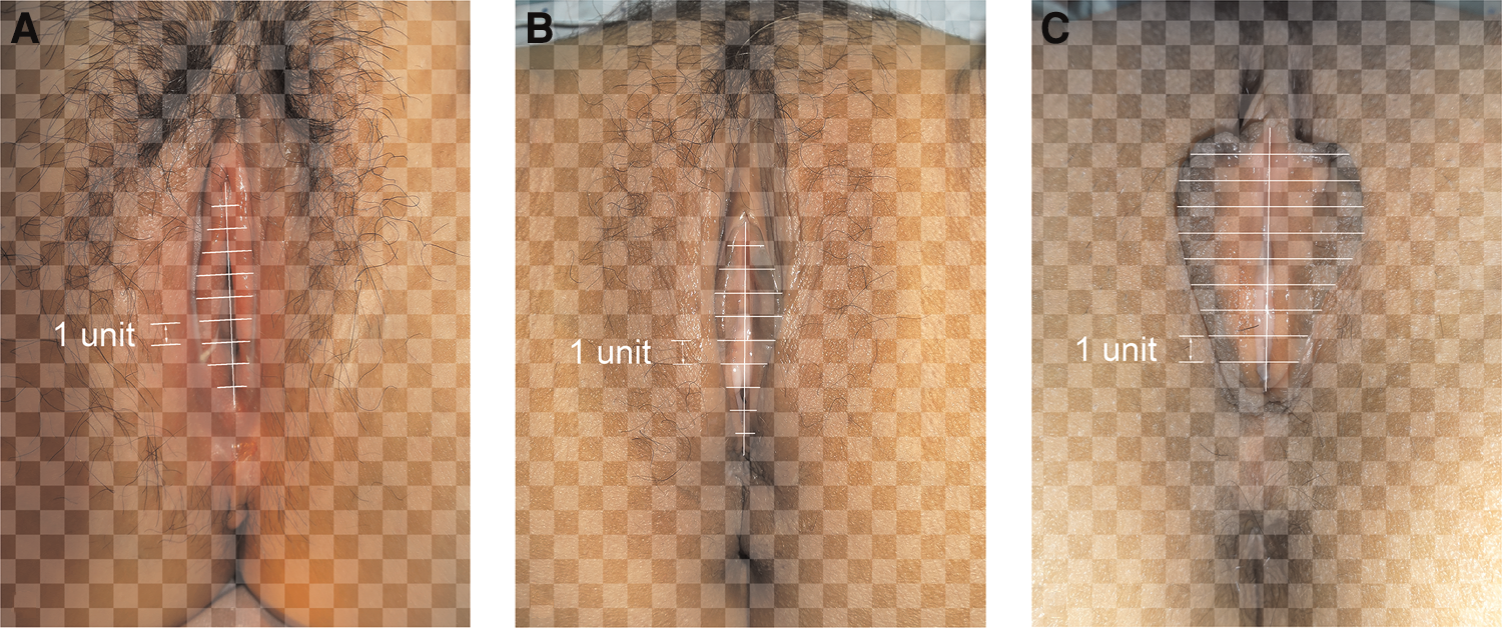
(**A**) “Small type” labia minora; (**B**) “willow leaf type” labia minora; (**C**) “butterfly wing type” labia minora.

**Figure 2:**
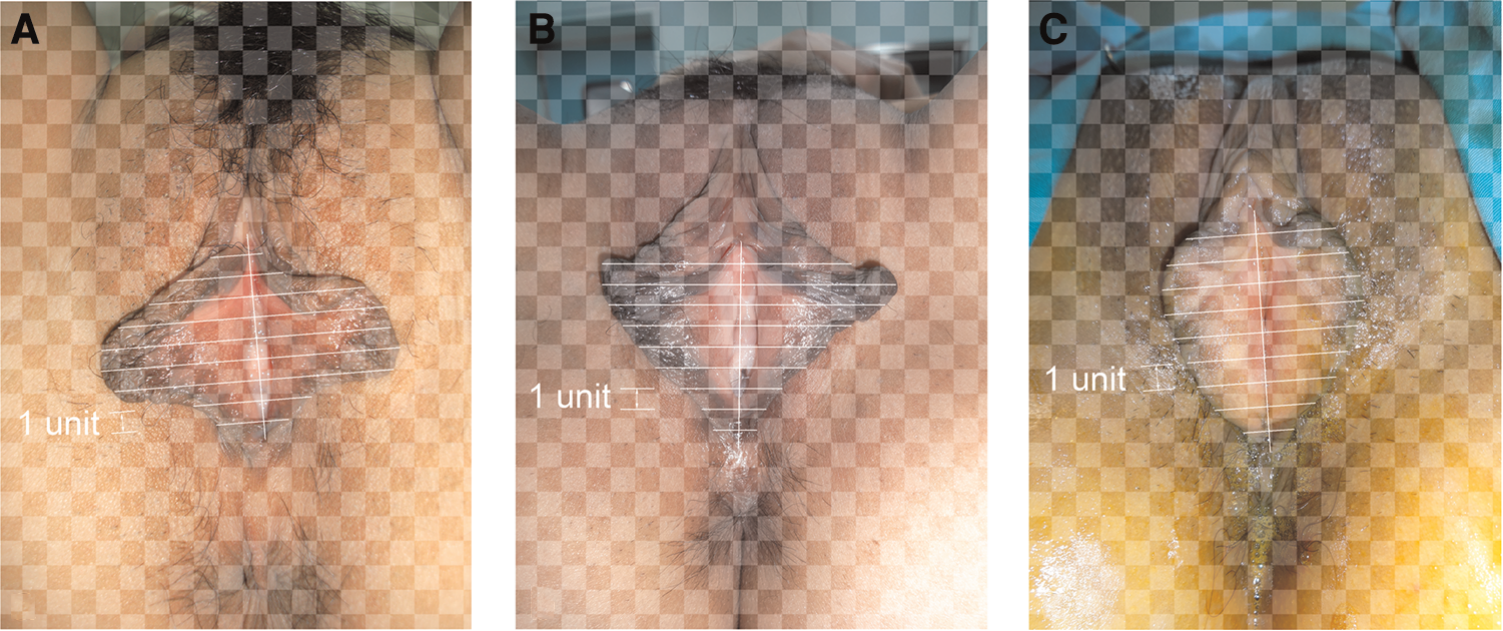
(**A**) “Protruding type” labia minora; (**B**) “rhombus type” labia minora; (**C**) “fan-shaped type” labia minora.

**Figure 3:**
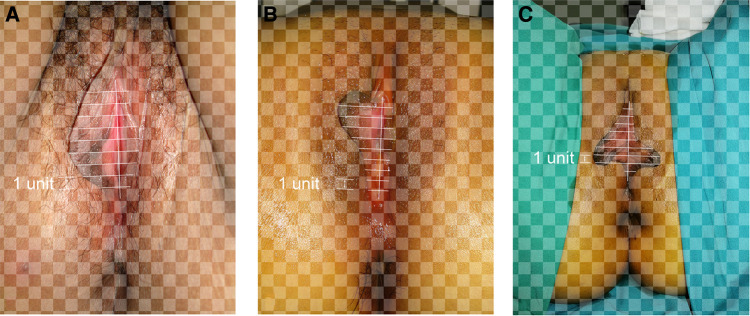
(**A–C**) Various types of bilateral labia minora asymmetry.

**Figure 4:**
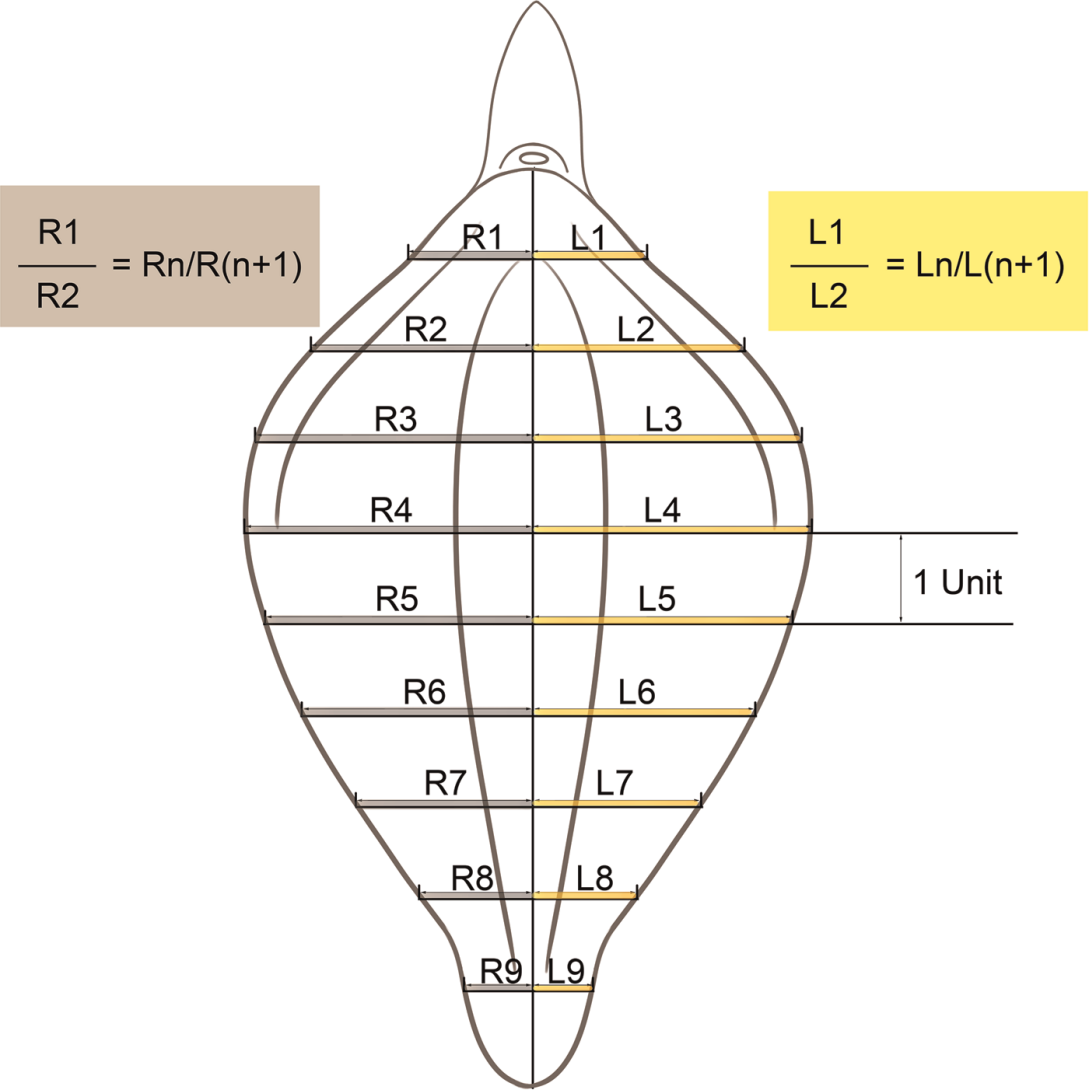
Graphical representation of the measurements performed on the labia minora.

**Figure 5:**
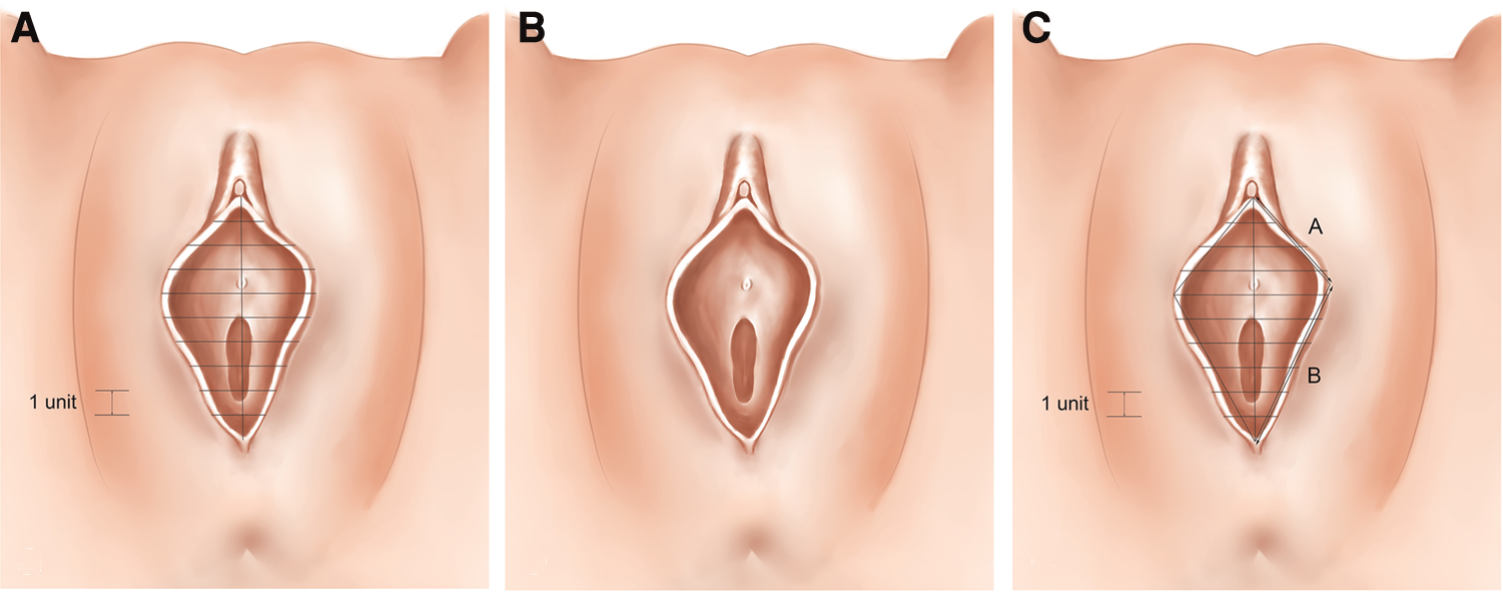
(**A–C**) Simulation of the average shape of labia minora in 400 patients (based on the mean values of L1–L9 and R1–R9).

Photographs of the 400 patients were reviewed retrospectively. The labia minora were naturally tiled laterally without traction when photographed. The longitudinal axis of the vaginal vestibule was marked into ten equal segments using Adobe Photoshop software. The length of one segment was set as one unit for measurement and plumb lines were made respectively (Figures 1–3). The well-marked photographs of the labia minora were then measured using ImageJ software. The vertical dimensions between the contour line of the labia minora and the longitudinal axis of the vaginal vestibule were calculated. The measured segments of the left side were named *Ln* (*n* = 1–9) and the measured segments of the right side were named *Rn* (*n* = 1–9) (Figure 4). In general, *L1–L9* represents a series of adjacent line segments on the left and R1–R9 represents a series of adjacent line segments on the right (Figure 4). Moreover, the ratio between adjacent line segments was calculated. *L1/L2*, *L2/L3*, *L3/L4*, *L4/L5*, *L5/L6*, *L7/L8*, and *L8/L9* were abbreviated as *Ln/L(n + 1)* (Figure 4). The same applied to *Rn/R(n + 1)*. The simulated graph of the average shape of the labia minora for adult Chinese women was constructed based on the mean values of *Ln* and *Rn* (Figure 5). Levene's test for equality of variances was performed before making comparisons between groups. Statistical significance was defined as *P*-value <0.05 (Supplementary Files 1–3).

## Results

### Measurements of *L1–L9* and *R1–R9*

The photographic measurement method used on various types of labia minora was shown in Figures 1–3. The maximum values among *L1–L9* and *R1–R9* in 137 patients (34.25%) fell into the range of *L1–L3* or *R1–R3*. For these patients, the prominent portions of the labia minora were the anterior parts. The maximum values of 187 patients (46.75%) fell into the range of *L4–L6* or *R4–R6*, and the prominent parts were the middle part of the labia minora. 76 patients (19%) had maximum values in the range of *L7–L9* or *R7–R9*. Thus, the anteriorly and medially protruding labia minora were more common than the posteriorly protruding labia minora. Based on the mean values of *L1–L9* and *R1–R9* in the 400 patients (Table 1), the average shape of the labia minora was constructed (Figure 5). The ratio of the posterior labial distance and the anterior labial distance was 1.498 (7.183/4.794) on the left side and 1.335 (6.942/5.200) on the right side. The former one was closer to the golden ratio (1.618:1).

**Table 1:** Measurements of L1–L9 and R1–R9.

	L1	L2	L3	L4	L5	L6	L7	L8	L9
Mean	1.137	2.314	3.039	3.333	3.196	2.784	2.059	1.784	1.314
(Mean L1)/(Mean L2)	(Mean L2)/(Mean L3)	(Mean L3)/(Mean L4)	(Mean L4)/(Mean L5)	(Mean L5)/(Mean L6)	(Mean L6)/(Mean L7)	(Mean L7)/(Mean L8)	(Mean L8)/(Mean L9)		
0.491	0.761	0.912	1.043	1.148	1.352	1.154	1.358		
	R1	R2	R3	R4	R5	R6	R7	R8	R9
Mean	0.941	2.235	3.059	3.078	2.843	2.314	1.765	1.412	0.941
(Mean R1)/(Mean R2)	(Mean R2)/(Mean R3)	(Mean R3)/(Mean R4)	(Mean R4)/(Mean R5)	(Mean R5)/(Mean R6)	(Mean R6)/(Mean R7)	(Mean R7)/(Mean R8)	(Mean R8)/(Mean R9)		
0.421	0.731	0.994	1.083	1.229	1.311	1.25	1.5		
	L1/L2		L2/L3	L3/L4	L4/L5	L5/L6	L6/L7	L7/L8	L8/L9
Mean [*Ln/L(n + 1)*]	0.524	0.763	0.916	1.067	1.176	1.47	1.23	1.448	
	R1/R2		R2/R3	R3/R4	R4/R5	R5/R6	R6/R7	R7/R8	R8/R9
Mean [*Rn/R(n + 1)*]	0.438	0.729	1.017	1.133	1.329	1.576	1.339	1.59	

### Association between the shape of labia minora and age

The average age of the included patients was 30.53 years, ranging from 18 to 70 years (Supplementary File 4). The 18–30 years was the main subgroup (Group A, *n* = 220, 55%), while others included the 31–40 years (Group B, *n* = 141, 35.25%), 41–50 years (Group C, *n* = 33, 8.25%), and ≥51 years (Group D, *n* = 6, 1.5%) (Table 2). Comparing the mean values of *Ln* and *Rn* in the four age groups revealed that the mean values gradually decreased with age. Independent-samples *t*-test was performed independently among groups from *L1* to *L9* (Supplementary File 1). Most groups showed no statistically significant differences (*P*-value >0.05), except for two subgroups [Group A (L9): Group B (L9), *P*-value = 0.010; Group B (L8): Group D (L8), *P*-value = 0.030, as shown in Table 2).

**Table 2:** Comparison between different age groups.

** **	Group A	Group B	Group C		Group D	All ages
Age	18–30 years	31–40 years	41–50 years		≥51 years	
** **	*n *= 220 (35.25%)	*n *= 141 (35.25%)	*n *= 33 (8.25%)		*n *= 6 (1.5%)	*n *= 400
Clinical measurements of the labia minora (mean value)						
L1	1.182	1.086	1.088		1.012	1.137
L2	2.358	2.289	2.221		1.904	2.314
L3	3.129	2.930	2.931		2.924	3.039
L4	3.425	3.243	3.256		2.615	3.333
L5	3.254	3.137	3.223		2.441	3.196
L6	2.819	2.723	2.960		2.083	2.784
L7	2.100	2.121	2.264		1.381	2.059
L8	1.725	1.900	1.828		1.085	1.784
L9	1.262	1.452	1.159		0.908	1.314
R1	0.962	0.913	0.932		0.901	0.941
R2	2.319	2.141	2.080		2.231	2.235
R3	3.126	3.012	2.791		3.211	3.059
R4	3.077	3.165	2.841		2.473	3.078
R5	2.879	2.892	2.508		2.324	2.843
R6	2.322	2.355	2.215		1.685	2.314
R7	1.749	1.861	1.625		0.992	1.765
R8	1.412	1.455	1.364		0.796	1.412
R9	0.946	0.958	0.910		0.586	0.941
Mean	2.225	2.202	2.122		1.753	2.197
** **	Group A: Group B	Group B: Group C	Group C: Group D	Group A: Group C	Group A: Group D	Group B: Group D
** **	*P*-value	*P*-value	*P*-value	*P*-value	*P*-value	*P*-value
L1	0.246	0.985	0.723	0.503	0.562	0.798
L2	0.612	0.775	0.496	0.560	0.350	0.420
L3	0.147	0.998	0.990	0.421	0.688	0.989
L4	0.141	0.950	0.163	0.446	0.078	0.125
L5	0.323	0.688	0.088	0.878	0.052	0.106
L6	0.354	0.221	0.074	0.446	0.045	0.080
L7	0.296	0.519	0.116	0.326	0.078	0.071
L8	0.079	0.705	0.063	0.539	0.056	0.030*
L9	0.010*	0.043	0.265	0.360	0.129	0.071
R1	0.442	0.855	0.881	0.790	0.797	0.957
R2	0.144	0.765	0.723	0.274	0.846	0.822
R3	0.368	0.332	0.381	0.122	0.849	0.667
R4	0.488	0.170	0.372	0.218	0.125	0.153
R5	0.919	0.092	0.661	0.062	0.180	0.226
R6	0.776	0.528	0.211	0.581	0.107	0.135
R7	0.366	0.320	0.117	0.534	0.065	0.075
R8	0.684	0.628	0.087	0.787	0.096	0.087
R9	0.863	0.693	0.132	0.747	0.119	0.128

* *P* < 0.05.

### Association between the shape of labia minora and the number of births

The average number of births of the included patients was 0.55. Patients were divided into three groups, including nulliparous women (Group 1, *n* = 243, 60.75%), primiparous women (Group 2, *n* = 99, 24.75%), and multiparous women (Group 3, *n* = 58, 14.5%) (Table 3). Based on the mean values of *Ln* and *Rn* for Group 1 to Group 3, the measured values of the labia minora in Group 2 (primiparous women) were the largest, followed by Group 1 (nulliparous women) and Group 3 (multiparous women) (Table 3). Independent sample *t*-tests were also performed for each group (Supplementary File 2).

**Table 3:** Comparison between different groups based on the number of births.

** **	Group 1	Group 2	Group 3	All
Number of births	0	1	≥2	** **
** **	*n* = 243 (60.75%)	*n* = 99 (24.75%)	*n* = 58 (14.5%)	*n* = 400
Clinical measurements of the labia minora (mean value)				
L1	1.167	1.125	1.035	1.137
L2	2.319	2.278	2.355	2.314
L3	3.059	3.055	2.928	3.039
L4	3.337	3.442	3.126	3.333
L5	3.186	3.314	3.034	3.196
L6	2.773	2.854	2.708	2.784
L7	2.035	2.070	2.140	2.059
L8	1.777	1.726	1.913	1.784
L9	1.290	1.365	1.328	1.314
R1	0.966	0.937	0.842	0.941
R2	2.273	2.138	2.240	2.235
R3	3.112	3.046	2.863	3.059
R4	3.101	3.178	2.810	3.078
R5	2.883	2.873	2.626	2.843
R6	2.305	2.413	2.182	2.314
R7	1.710	1.898	1.767	1.765
R8	1.371	1.402	1.604	1.412
R9	0.940	0.928	0.969	0.941
Mean	2.200	2.224	2.137	2.197
** **	Group 1: Group 2	Group 2: Group 3	Group 1: Group 3
** **	*P*-value	*P*-value	*P*-value
L1	0.651	0.448	0.220
L2	0.778	0.713	0.842
L3	0.978	0.538	0.492
L4	0.450	0.085	0.202
L5	0.342	0.114	0.336
L6	0.488	0.392	0.636
L7	0.769	0.711	0.556
L8	0.635	0.212	0.333
L9	0.367	0.749	0.697
R1	0.685	0.258	0.155
R2	0.313	0.571	0.846
R3	0.637	0.328	0.150
R4	0.571	0.042	0.067
R5	0.945	0.175	0.114
R6	0.414	0.210	0.414
R7	0.174	0.516	0.721
R8	0.782	0.239	0.105
R9	0.864	0.690	0.752

Group 1, Group 2, and Group 3 did not differ statistically significantly (*P*-value >0.05, Table 3). The detailed statistical data and independent samples *t*-test were shown in Supplementary File 2.

### Association between the shape of the labia minora and procedures going to perform

The procedures performed in 223 patients (Group I, 55.75%) included unilateral or bilateral labiaplasty. A total of 177 patients (Group II, 44.2%) did not undergo labiaplasty (Supplementary File 4). Data from Group I and Group II were also compared (Supplementary File 3). Although the values and means of *Ln* and *Rn* were different in the two groups, there were no statistical differences (*P*-value >0.05, Table 4).

**Table 4:** Comparison between different groups based on surgical information.

	Group I	Group II	All
Whether labiaplasty has been performed	Yes	No	
	*n* = 223 (55.75%)	*n* = 177 (44.2%)	*n* = 400
Clinical measurements of the labia minora (mean value)			Group I: Group II (*P*-value)
L1	1.146	1.126	0.786
L2	2.326	2.299	0.825
L3	3.02	3.063	0.734
L4	3.343	3.319	0.834
L5	3.249	3.128	0.273
L6	2.841	2.712	0.191
L7	2.113	1.99	0.234
L8	1.846	1.705	0.13
L9	1.328	1.297	0.65
R1	0.948	0.932	0.788
R2	2.187	2.294	0.343
R3	3.034	3.091	0.626
R4	3.095	3.057	0.738
R5	2.884	2.792	0.413
R6	2.312	2.316	0.968
R7	1.727	1.813	0.452
R8	1.396	1.433	0.705
R9	0.917	0.971	0.381
Mean	2.206	2.186	

## Discussion

The purpose of the study is to investigate the morphological features of the labia minora in adult women who had never had genital surgery before the vulvar measurement. After a graphic-to-digital and digital-to-graphic conversion, the average shape of the 400 included patients was constructed (Figure 5). Meanwhile, data were further analyzed to explore the association between the labia minora morphology and factors such as age, parity, and whether they planned to have labia minora surgery.

Female genital cosmetic surgery, particularly labiaplasty, is gaining in popularity (17). However, there has been no unified “aesthetic gold standard” for the shape of the labia minora to date. It is thought that the aesthetics of the female vulva is not determined by one or two structures but by the vulvar region as a whole. Therefore, an aesthetically pleasing labia minora must coordinate with adjacent structures (18). Most previous studies on the size of the labia minora have focused only on the width and length of the labia minora (8, 13, 18, 19). In this study, the shape characteristics of the labia minora were converted into measurable values which can be used for comparative analysis. The values of *Ln*, *Rn*, *Ln/L(n + 1)*, and *Rn/R(N + 1)* reflected the contour of the labia minora and the labia minora characteristics.

The shape features of the labia minora in Chinese adult women could provide a useful supplement to the field of female genital plastic surgery. Simplifying the contours of a body part or organ into geometric figures is a common method for biomorphology (20, 21). In this study, the method used to measure labia minora draws on this strategy. The unit length was set as one-tenth of the longitudinal length of the vaginal vestibule in each patient, making it feasible to compare the shape of the labia minora between patients. The larger the *Ln* and *Rn* values, the wider the shape of the labia minora. In contrast, the smaller the *Ln* and *Rn* values, the thinner the shape of the labia minora. *L1/L2*, *L2/L3*, *L3/L4*, *L4/L5*, *L5/L6*, *L7/L8*, and *L8/L9* represent the trend in the distance between the left labia minora edge and the median axis. Same for *Rn/R(n + 1)*. When *Ln/L(n + 1)* or *Rn/R(n + 1)* value is 1, it means that the two adjacent line segments are equal. When *Ln/L(n + 1)* and *Rn/R(n + 1)* are greater than 1, the larger the value, the larger the gap between adjacent line segments. On the contrary, when the ratio is less than 1, the smaller the value, the larger the gap between adjacent line segments. Overall, *Ln/L(n + 1)* and *Rn/R(n + 1)* determined the protrusion degree of the labia minora when observing the vulva as a whole (Table 1). The average shape of the labia minora in the 400 included patients was constructed based on the mean values of *L1–L9* and *R1–R9* (Figure 5; Table 1). To some extent, it could represent the general shape of the labia minora in Chinese adult females. The simulated outline of the labia minora was close to a “butterfly wing shape” (Figure 5).

The golden rule is widely used in plastic surgery as a generally accepted aesthetic standard. The labia minora could also refer to the golden ratio to achieve visual beauty (22). For aesthetically pleasing labia minora, there is a golden ratio (1:1.618) between the anterior and posterior labial distances of the labia minora (23). In the present study, the ratio of the posterior labial distance (Line B) and the anterior labial distance (Line A) was 1.498 (7.183/4.794) on the left side and 1.335 (6.942/5.200) on the right side (Figure 5), with the ratio of left labia minora closer to the golden ratio.

The labia minora seemed to acquire a thinner shape with increasing age (Table 2). Nevertheless, no significant differences were found between most age groups when *Ln* and *Rn* values were compared, except for two subgroups (Table 2). As a result, it appears that there was no significant relationship between age and labia minora measurements, consistent with previous studies. For instance, Lloyd et al. reported no significant associations between the size of labia minora and age, parity, hormone use, or sexual history (4). In terms of parity, primiparous women (Group 2) had the largest *Ln* and *Rn* values (Table 3), implying that the labia minora seemed to be wider for this patient group. In comparison, the smallest value corresponded to Group 3 (number of births ≥2), which means that for females with two and more births, their labia minora appeared to be thinner (Table 3). However, the difference in mean values between the three groups was quite small, and no significant differences were found (*P*-value >0.05) (Supplementary File 2, Table 3). Patients who planned to undergo labiaplasty usually have a strong subjective desire since labiaplasty is a cosmetic procedure (24, 25). It was mentioned in certain studies that the size of the labia minora was associated with patients’ subjective perception (12, 14, 26). In this study, the association between whether labiaplasty was performed and the shape characteristics of the labia minora was also analyzed (Supplementary File 3). For patients who underwent unilateral or bilateral labia minora surgery, the values of *Ln* and *Rn* were larger (Table 4). Their labia minora appeared to be wider and more prominent than patients who did not undergo labiaplasty. However, all *P*-values were greater than 0.05 and there was no significant difference between Group I and Group II (Table 4). It seems to contradict other previous studies (12, 14, 26). While the exact cause is not known, we speculate that this may be due to uneven grouping and a small sample size in a single medical center. Moreover, the labia minora surgery is not only determined by the shape of the labia minora but also the associated physical symptoms and the patient's wishes. There are many patients seeking labiaplasty who have no physical symptoms, such as hygiene problems, tugging during intercourse, uncomfortable wearing tight clothing, pain during intercourse, erosions, etc. (1, 2, 27). They seek labiaplasty to achieve their “ideal labia minora”. Therefore, patients scheduled for labiaplasty may not have more hypertrophied labia minora than those who were not going to have labiaplasty.

The vaginal vestibule, as an inseparable structure from the labia minora, refers to the area between the two sides of the labia minora (11). The aesthetics of the labia minora is not only associated with the specific value of the width of the labia minora (28). Even if two patients have the same labia minora width, if their vaginal vestibule lengths differ, the shape and aesthetics of the labia minora will also differ. The labia minora and the adjacent vestibule should therefore be regarded as a whole (19, 29). Various factors such as weight and height may affect measurements of labia minora, so a direct comparison of the labia minora width could be uncomprehensive (15, 30, 31). The measurement strategy in the study is helpful to reveal the overall shape features. To our knowledge, no previous studies have quantified the shape features of the labia minora. Related studies usually focused on subjective perceptions, measurements of local structure, and rough generalizations. Herein, we further grouped and analyzed 400 patients at three levels (age, parity, and whether the labiaplasty going to perform). Independent samples *t*-tests were performed to explore whether the differences between the groups were significant.

Currently available classifications for labia protrusion are based on the distance between the lateral edge of the labia minora and the labia majora, or the distance between the base of the labia minora and the distalmost tip (19). Our finding could help plastic surgeons gain a direct visual cognition and understanding of the labia minora in Chinese adult women. It would be helpful to provide more guidance to plastic surgeons. Even if two patients have the same labia minora width, if the shape of the labia minora differs, the aesthetics of the labia minora and patients’ perceptions will also differ. We believe that the study based on quantifiable data and analysis is important for the further exploration of labia minora morphology.

Despite the fact that the study was only based on retrospective photographs, the combination of in-depth data analysis by image processing software gives this study an advantage over other studies. Although the photographs were not taken from the same distance all the time, the relative sizes of each part of the labia minora were not affected when calculating the values of *Ln*, *Rn*, *Rn/R(n + 1)*, and *Ln/L(n + 1)*. Another shortcoming of the study is that although we included patients with different clinical characteristics and surgical plans, bias due to population selection still exists. Since patients were obtained from a single center, the data presented here might be limited and caution is warranted when interpreting our findings. In the future, to correlate morphologic characteristics with surgical outcomes, clinical applications may be required to establish its utility. More large-scale studies from multi-centers are required to further validate our results. New criteria and references for labial morphology may develop subsequently.

## Conclusion

The average shape of labia minora in 400 Chinese adult women could provide a visual reference to plastic surgeons. The shape of labia minora was not significantly associated with age, parity, or whether or not the patient was going to undergo labiaplasty. The shape characteristics of the labia minora were converted into numerical values and a new method was provided for measuring and evaluating the shape of the labia minora. Collectively, our findings could be useful for a better understanding of the morphology of labia minora.

## Data Availability

The original contributions presented in the study are included in the article/Supplementary Material, further inquiries can be directed to the corresponding author/s.
