# Supplementary material for: Quantitative analysis of the labia minora morphology in 400 Chinese women: A new method for assessing the shape of the labia minora
Source: Front Surg. 2023 Jan 6;9:961247. doi: 10.3389/fsurg.2022.961247 (PMC9852508; doi:10.3389/fsurg.2022.961247)
Supplement: Supplementary file 1 [file Table1.docx]

**Supplementary file 1. Group statistics and independent samples t-tests for the four age groups.**

| **Group "18~30 age" & Group "31~40 age"** | | | |  |  |
| --- | --- | --- | --- | --- | --- |
|  | Age | N | Mean | Std. Deviation | Std. Error Mean |
| L1 | 18~30 | 219 | 1.1819 | 0.77218 | 0.05218 |
|  | 31~40 | 141 | 1.0858 | 0.75747 | 0.06379 |
| L2 | 18~30 | 219 | 2.3575 | 1.26894 | 0.08575 |
|  | 31~40 | 141 | 2.2887 | 1.23796 | 0.10426 |
| L3 | 18~30 | 219 | 3.1287 | 1.31405 | 0.08879 |
|  | 31~40 | 141 | 2.9304 | 1.18397 | 0.09971 |
| L4 | 18~30 | 219 | 3.4248 | 1.19665 | 0.08086 |
|  | 31~40 | 141 | 3.2429 | 1.05469 | 0.08882 |
| L5 | 18~30 | 219 | 3.2539 | 1.08755 | 0.07349 |
|  | 31~40 | 141 | 3.1368 | 1.10835 | 0.09334 |
| L6 | 18~30 | 219 | 2.8186 | 0.9573 | 0.06469 |
|  | 31~40 | 141 | 2.7231 | 0.94422 | 0.07952 |
| L7 | 18~30 | 219 | 2.0096 | 0.93286 | 0.06304 |
|  | 31~40 | 141 | 2.1211 | 1.0674 | 0.08989 |
| L8 | 18~30 | 219 | 1.725 | 0.87824 | 0.05935 |
|  | 31~40 | 141 | 1.8998 | 0.97717 | 0.08229 |
| L9 | 18~30 | 219 | 1.2619 | 0.6078 | 0.04107 |
|  | 31~40 | 141 | 1.452 | 0.78096 | 0.06577 |
| R1 | 18~30 | 219 | 0.9619 | 0.62187 | 0.04202 |
|  | 31~40 | 141 | 0.9125 | 0.54636 | 0.04601 |
| R2 | 18~30 | 219 | 2.3188 | 1.18186 | 0.07986 |
|  | 31~40 | 141 | 2.1405 | 1.0401 | 0.08759 |
| R3 | 18~30 | 219 | 3.1256 | 1.15962 | 0.07836 |
|  | 31~40 | 141 | 3.0118 | 1.18657 | 0.09993 |
| R4 | 18~30 | 219 | 3.077 | 1.02719 | 0.06941 |
|  | 31~40 | 141 | 3.165 | 1.25953 | 0.10607 |
| R5 | 18~30 | 219 | 2.8792 | 1.07474 | 0.07262 |
|  | 31~40 | 141 | 2.8917 | 1.21212 | 0.10208 |
| R6 | 18~30 | 219 | 2.322 | 1.03267 | 0.06978 |
|  | 31~40 | 141 | 2.3555 | 1.16763 | 0.09833 |
| R7 | 18~30 | 219 | 1.7486 | 1.07372 | 0.07256 |
|  | 31~40 | 141 | 1.8614 | 1.27211 | 0.10713 |
| R8 | 18~30 | 219 | 1.412 | 0.96939 | 0.06551 |
|  | 31~40 | 141 | 1.4552 | 1.00544 | 0.08467 |
| R9 | 18~30 | 219 | 0.9463 | 0.6049 | 0.04088 |
|  | 31~40 | 141 | 0.9578 | 0.63681 | 0.05363 |

| **Group "18~30 age" & Group "31~40 age"** | | | | | | | | | | |
| --- | --- | --- | --- | --- | --- | --- | --- | --- | --- | --- |
|  |  | Levene's Test for Equality of Variances | | t-test for Equality of Means | |  |  |  |  |  |
|  |  | F | Sig. | t | df | Sig. (2-tailed) | Mean Difference | Std.Error Difference | 95% Confidence interval of the Difference | |
|  |  |  |  |  |  |  |  |  | Lower | Upper |
| L1 | Equal variances assumed | 0.895 | 0.345 | 1.162 | 358 | 0.246 | 0.09615 | 0.08276 | -0.0666 | 0.2589 |
|  | Equal variances not assumed |  |  | 1.167 | 302.927 | 0.244 | 0.09615 | 0.08241 | -0.06602 | 0.25833 |
| L2 | Equal variances assumed | 0.856 | 0.356 | 0.507 | 358 | 0.612 | 0.06885 | 0.13571 | -0.19805 | 0.33574 |
|  | Equal variances not assumed |  |  | 0.51 | 304.104 | 0.61 | 0.06885 | 0.13499 | -0.19678 | 0.33448 |
| L3 | Equal variances assumed | 0.757 | 0.385 | 1.452 | 358 | 0.147 | 0.19827 | 0.13656 | -0.0703 | 0.46684 |
|  | Equal variances not assumed |  |  | 1.485 | 320.613 | 0.139 | 0.19827 | 0.13351 | -0.06441 | 0.46095 |
| L4 | Equal variances assumed | 0.461 | 0.498 | 1.474 | 358 | 0.141 | 0.1819 | 0.12344 | -0.06086 | 0.42466 |
|  | Equal variances not assumed |  |  | 1.514 | 324.907 | 0.131 | 0.1819 | 0.12012 | -0.0544 | 0.41821 |
| L5 | Equal variances assumed | 0.045 | 0.832 | 0.99 | 358 | 0.323 | 0.11711 | 0.11831 | -0.11556 | 0.34979 |
|  | Equal variances not assumed |  |  | 0.986 | 294.655 | 0.325 | 0.11711 | 0.1188 | -0.11669 | 0.35092 |
| L6 | Equal variances assumed | 0.047 | 0.828 | 0.929 | 358 | 0.354 | 0.09549 | 0.10281 | -0.1067 | 0.29769 |
|  | Equal variances not assumed |  |  | 0.932 | 301.747 | 0.352 | 0.09549 | 0.10251 | -0.10622 | 0.29721 |
| L7 | Equal variances assumed | 3.153 | 0.077 | -1.046 | 358 | 0.296 | -0.1115 | 0.10664 | -0.32123 | 0.09822 |
|  | Equal variances not assumed |  |  | -1.016 | 269.669 | 0.311 | -0.1115 | 0.10979 | -0.32766 | 0.10465 |
| L8 | Equal variances assumed | 2.234 | 0.136 | -1.763 | 358 | 0.079 | -0.17481 | 0.09914 | -0.36978 | 0.02016 |
|  | Equal variances not assumed |  |  | -1.723 | 275.612 | 0.086 | -0.17481 | 0.10146 | -0.37454 | 0.02493 |
| L9 | Equal variances assumed | 2.268 | 0.133 | -2.586 | 358 | 0.01 | -0.19011 | 0.07351 | -0.33466 | -0.04555 |
|  | Equal variances not assumed |  |  | -2.452 | 246.417 | 0.015 | -0.19011 | 0.07754 | -0.34283 | -0.03738 |
| R1 | Equal variances assumed | 0.242 | 0.623 | 0.77 | 358 | 0.442 | 0.04937 | 0.06408 | -0.07665 | 0.17539 |
|  | Equal variances not assumed |  |  | 0.792 | 325.511 | 0.429 | 0.04937 | 0.06231 | -0.07322 | 0.17196 |
| R2 | Equal variances assumed | 1.484 | 0.224 | 1.463 | 358 | 0.144 | 0.17826 | 0.12185 | -0.06138 | 0.4179 |
|  | Equal variances not assumed |  |  | 1.504 | 325.193 | 0.134 | 0.17826 | 0.11853 | -0.05493 | 0.41145 |
| R3 | Equal variances assumed | 0.102 | 0.75 | 0.901 | 358 | 0.368 | 0.11382 | 0.12635 | -0.13467 | 0.36231 |
|  | Equal variances not assumed |  |  | 0.896 | 293.777 | 0.371 | 0.11382 | 0.12699 | -0.1361 | 0.36374 |
| R4 | Equal variances assumed | 6.651 | 0.01 | -0.725 | 358 | 0.469 | -0.08794 | 0.12134 | -0.32657 | 0.15069 |
|  | Equal variances not assumed |  |  | -0.694 | 255.486 | 0.488 | -0.08794 | 0.12676 | -0.33757 | 0.1617 |
| R5 | Equal variances assumed | 0.724 | 0.396 | -0.102 | 358 | 0.919 | -0.01246 | 0.12206 | -0.2525 | 0.22759 |
|  | Equal variances not assumed |  |  | -0.099 | 272.722 | 0.921 | -0.01246 | 0.12528 | -0.25909 | 0.23418 |
| R6 | Equal variances assumed | 0.485 | 0.487 | -0.285 | 358 | 0.776 | -0.03347 | 0.11742 | -0.26438 | 0.19744 |
|  | Equal variances not assumed |  |  | -0.278 | 272.184 | 0.782 | -0.03347 | 0.12058 | -0.27085 | 0.20391 |
| R7 | Equal variances assumed | 1.549 | 0.214 | -0.905 | 358 | 0.366 | -0.11285 | 0.12475 | -0.35818 | 0.13249 |
|  | Equal variances not assumed |  |  | -0.872 | 262.427 | 0.384 | -0.11285 | 0.12939 | -0.36762 | 0.14193 |
| R8 | Equal variances assumed | 0.041 | 0.84 | -0.407 | 358 | 0.684 | -0.0432 | 0.10621 | -0.25207 | 0.16567 |
|  | Equal variances not assumed |  |  | -0.404 | 290.826 | 0.687 | -0.0432 | 0.10705 | -0.2539 | 0.1675 |
| R9 | Equal variances assumed | 0.035 | 0.851 | -0.172 | 358 | 0.863 | -0.01148 | 0.06668 | -0.14262 | 0.11965 |
|  | Equal variances not assumed |  |  | -0.17 | 287.578 | 0.865 | -0.01148 | 0.06743 | -0.1442 | 0.12124 |

| **Group "31~40 age" & Group "41~50 age"** | | | |  |  |
| --- | --- | --- | --- | --- | --- |
|  | Age | N | Mean | Std. Deviation | Std. Error Mean |
| L1 | 31~40 | 141 | 1.0858 | 0.75747 | 0.06379 |
|  | 41~50 | 33 | 1.0884 | 0.54421 | 0.09473 |
| L2 | 31~40 | 141 | 2.2887 | 1.23796 | 0.10426 |
|  | 41~50 | 33 | 2.2211 | 1.13281 | 0.1972 |
| L3 | 31~40 | 141 | 2.9304 | 1.18397 | 0.09971 |
|  | 41~50 | 33 | 2.931 | 1.30134 | 0.22653 |
| L4 | 31~40 | 141 | 3.2429 | 1.05469 | 0.08882 |
|  | 41~50 | 33 | 3.2559 | 1.10271 | 0.19196 |
| L5 | 31~40 | 141 | 3.1368 | 1.10835 | 0.09334 |
|  | 41~50 | 33 | 3.2227 | 1.09303 | 0.19027 |
| L6 | 31~40 | 141 | 2.7231 | 0.94422 | 0.07952 |
|  | 41~50 | 33 | 2.9602 | 1.20404 | 0.2096 |
| L7 | 31~40 | 141 | 2.1211 | 1.0674 | 0.08989 |
|  | 41~50 | 33 | 2.2639 | 1.42156 | 0.24746 |
| L8 | 31~40 | 141 | 1.8998 | 0.97717 | 0.08229 |
|  | 41~50 | 33 | 1.8278 | 1.0026 | 0.17453 |
| L9 | 31~40 | 141 | 1.452 | 0.78096 | 0.06577 |
|  | 41~50 | 33 | 1.1591 | 0.54213 | 0.09437 |
| R1 | 31~40 | 141 | 0.9125 | 0.54636 | 0.04601 |
|  | 41~50 | 33 | 0.9316 | 0.50826 | 0.08848 |
| R2 | 31~40 | 141 | 2.1405 | 1.0401 | 0.08759 |
|  | 41~50 | 33 | 2.0802 | 1.04538 | 0.18198 |
| R3 | 31~40 | 141 | 3.0118 | 1.18657 | 0.09993 |
|  | 41~50 | 33 | 2.7913 | 1.0994 | 0.19138 |
| R4 | 31~40 | 141 | 3.165 | 1.25953 | 0.10607 |
|  | 41~50 | 33 | 2.8406 | 1.01444 | 0.17659 |
| R5 | 31~40 | 141 | 2.8917 | 1.21212 | 0.10208 |
|  | 41~50 | 33 | 2.5076 | 0.9839 | 0.17127 |
| R6 | 31~40 | 141 | 2.3555 | 1.16763 | 0.09833 |
|  | 41~50 | 33 | 2.2153 | 1.04137 | 0.18128 |
| R7 | 31~40 | 141 | 1.8614 | 1.27211 | 0.10713 |
|  | 41~50 | 33 | 1.6247 | 1.00503 | 0.17495 |
| R8 | 31~40 | 141 | 1.4552 | 1.00544 | 0.08467 |
|  | 41~50 | 33 | 1.3639 | 0.82179 | 0.14306 |
| R9 | 31~40 | 141 | 0.9578 | 0.63681 | 0.05363 |
|  | 41~50 | 33 | 0.9104 | 0.53534 | 0.09319 |

| **Group "31~40 age" & Group "41~50 age"** | | | | | | | | | | |
| --- | --- | --- | --- | --- | --- | --- | --- | --- | --- | --- |
|  |  | Levene's Test for Equality of Variances | | t-test for Equality of Means | |  |  |  |  |  |
|  |  | F | Sig. | t | df | Sig. (2-tailed) | Mean Difference | Std.Error Difference | 95% Confidence interval of the Difference | |
|  |  |  |  |  |  |  |  |  | Lower | Upper |
| L1 | Equal variances assumed | 0.857 | 0.356 | -0.019 | 172 | 0.985 | -0.00267 | 0.13973 | -0.27847 | 0.27314 |
|  | Equal variances not assumed |  |  | -0.023 | 64.563 | 0.981 | -0.00267 | 0.11421 | -0.23079 | 0.22546 |
| L2 | Equal variances assumed | 0.001 | 0.977 | 0.287 | 172 | 0.775 | 0.06758 | 0.23575 | -0.39775 | 0.5329 |
|  | Equal variances not assumed |  |  | 0.303 | 51.469 | 0.763 | 0.06758 | 0.22306 | -0.38014 | 0.51529 |
| L3 | Equal variances assumed | 0.709 | 0.401 | -0.002 | 172 | 0.998 | -0.00057 | 0.23334 | -0.46115 | 0.46002 |
|  | Equal variances not assumed |  |  | -0.002 | 45.212 | 0.998 | -0.00057 | 0.24751 | -0.49901 | 0.49787 |
| L4 | Equal variances assumed | 0.002 | 0.965 | -0.063 | 172 | 0.95 | -0.01295 | 0.20571 | -0.41899 | 0.3931 |
|  | Equal variances not assumed |  |  | -0.061 | 46.68 | 0.951 | -0.01295 | 0.21151 | -0.43853 | 0.41263 |
| L5 | Equal variances assumed | 0.054 | 0.817 | -0.402 | 172 | 0.688 | -0.08589 | 0.21378 | -0.50787 | 0.33608 |
|  | Equal variances not assumed |  |  | -0.405 | 48.611 | 0.687 | -0.08589 | 0.21193 | -0.51188 | 0.34009 |
| L6 | Equal variances assumed | 2.961 | 0.087 | -1.228 | 172 | 0.221 | -0.23701 | 0.19293 | -0.61783 | 0.14381 |
|  | Equal variances not assumed |  |  | -1.057 | 41.677 | 0.296 | -0.23701 | 0.22417 | -0.68951 | 0.21549 |
| L7 | Equal variances assumed | 3.627 | 0.059 | -0.647 | 172 | 0.519 | -0.14282 | 0.22077 | -0.57858 | 0.29295 |
|  | Equal variances not assumed |  |  | -0.542 | 40.84 | 0.59 | -0.14282 | 0.26328 | -0.67459 | 0.38895 |
| L8 | Equal variances assumed | 0.045 | 0.831 | 0.379 | 172 | 0.705 | 0.07204 | 0.18989 | -0.30277 | 0.44685 |
|  | Equal variances not assumed |  |  | 0.373 | 47.276 | 0.711 | 0.07204 | 0.19296 | -0.31608 | 0.46016 |
| L9 | Equal variances assumed | 1.333 | 0.25 | 2.04 | 172 | 0.043 | 0.29288 | 0.14356 | 0.00951 | 0.57624 |
|  | Equal variances not assumed |  |  | 2.546 | 67.018 | 0.013 | 0.29288 | 0.11503 | 0.06328 | 0.52248 |
| R1 | Equal variances assumed | 0.257 | 0.613 | -0.182 | 172 | 0.855 | -0.01904 | 0.10432 | -0.22495 | 0.18688 |
|  | Equal variances not assumed |  |  | -0.191 | 50.8 | 0.849 | -0.01904 | 0.09973 | -0.21926 | 0.18119 |
| R2 | Equal variances assumed | 0.039 | 0.843 | 0.3 | 172 | 0.765 | 0.06034 | 0.20132 | -0.33704 | 0.45772 |
|  | Equal variances not assumed |  |  | 0.299 | 47.957 | 0.766 | 0.06034 | 0.20196 | -0.34574 | 0.46642 |
| R3 | Equal variances assumed | 0.751 | 0.387 | 0.974 | 172 | 0.332 | 0.22043 | 0.22642 | -0.22649 | 0.66734 |
|  | Equal variances not assumed |  |  | 1.021 | 50.961 | 0.312 | 0.22043 | 0.2159 | -0.21302 | 0.65387 |
| R4 | Equal variances assumed | 1.732 | 0.19 | 1.378 | 172 | 0.17 | 0.3244 | 0.23547 | -0.14039 | 0.78918 |
|  | Equal variances not assumed |  |  | 1.575 | 57.544 | 0.121 | 0.3244 | 0.206 | -0.08803 | 0.73682 |
| R5 | Equal variances assumed | 1.444 | 0.231 | 1.693 | 172 | 0.092 | 0.38413 | 0.22684 | -0.06362 | 0.83187 |
|  | Equal variances not assumed |  |  | 1.927 | 57.124 | 0.059 | 0.38413 | 0.19939 | -0.01512 | 0.78338 |
| R6 | Equal variances assumed | 0.963 | 0.328 | 0.633 | 172 | 0.528 | 0.14019 | 0.22146 | -0.29693 | 0.57731 |
|  | Equal variances not assumed |  |  | 0.68 | 52.561 | 0.5 | 0.14019 | 0.20623 | -0.27354 | 0.55392 |
| R7 | Equal variances assumed | 1.381 | 0.242 | 0.998 | 172 | 0.32 | 0.23676 | 0.23724 | -0.23152 | 0.70505 |
|  | Equal variances not assumed |  |  | 1.154 | 58.613 | 0.253 | 0.23676 | 0.20515 | -0.1738 | 0.64732 |
| R8 | Equal variances assumed | 0.64 | 0.425 | 0.485 | 172 | 0.628 | 0.0913 | 0.18833 | -0.28043 | 0.46304 |
|  | Equal variances not assumed |  |  | 0.549 | 56.756 | 0.585 | 0.0913 | 0.16624 | -0.24161 | 0.42422 |
| R9 | Equal variances assumed | 0.806 | 0.371 | 0.396 | 172 | 0.693 | 0.0474 | 0.11974 | -0.18895 | 0.28374 |
|  | Equal variances not assumed |  |  | 0.441 | 55.318 | 0.661 | 0.0474 | 0.10752 | -0.16805 | 0.26284 |

| **Group "41~50 age" & Group "51~70 age"** | | | |  |  |
| --- | --- | --- | --- | --- | --- |
|  | Age | N | Mean | Std. Deviation | Std. Error Mean |
| L1 | 41~50 | 33 | 1.0884 | 0.54421 | 0.09473 |
|  | 51~70 | 7 | 1.0119 | 0.31726 | 0.11991 |
| L2 | 41~50 | 33 | 2.2211 | 1.13281 | 0.1972 |
|  | 51~70 | 7 | 1.904 | 0.96841 | 0.36603 |
| L3 | 41~50 | 33 | 2.931 | 1.30134 | 0.22653 |
|  | 51~70 | 7 | 2.9237 | 1.7104 | 0.64647 |
| L4 | 41~50 | 33 | 3.2559 | 1.10271 | 0.19196 |
|  | 51~70 | 7 | 2.615 | 0.97054 | 0.36683 |
| L5 | 41~50 | 33 | 3.2227 | 1.09303 | 0.19027 |
|  | 51~70 | 7 | 2.4414 | 0.95663 | 0.36157 |
| L6 | 41~50 | 33 | 2.9602 | 1.20404 | 0.2096 |
|  | 51~70 | 7 | 2.0826 | 0.77619 | 0.29337 |
| L7 | 41~50 | 33 | 2.2639 | 1.42156 | 0.24746 |
|  | 51~70 | 7 | 1.3806 | 0.51235 | 0.19365 |
| L8 | 41~50 | 33 | 1.8278 | 1.0026 | 0.17453 |
|  | 51~70 | 7 | 1.0847 | 0.39554 | 0.1495 |
| L9 | 41~50 | 33 | 1.1591 | 0.54213 | 0.09437 |
|  | 51~70 | 7 | 0.9083 | 0.47806 | 0.18069 |
| R1 | 41~50 | 33 | 0.9316 | 0.50826 | 0.08848 |
|  | 51~70 | 7 | 0.9011 | 0.33978 | 0.12842 |
| R2 | 41~50 | 33 | 2.0802 | 1.04538 | 0.18198 |
|  | 51~70 | 7 | 2.2309 | 0.83816 | 0.3168 |
| R3 | 41~50 | 33 | 2.7913 | 1.0994 | 0.19138 |
|  | 51~70 | 7 | 3.211 | 1.3287 | 0.5022 |
| R4 | 41~50 | 33 | 2.8406 | 1.01444 | 0.17659 |
|  | 51~70 | 7 | 2.4731 | 0.74729 | 0.28245 |
| R5 | 41~50 | 33 | 2.5076 | 0.9839 | 0.17127 |
|  | 51~70 | 7 | 2.3236 | 1.09109 | 0.41239 |
| R6 | 41~50 | 33 | 2.2153 | 1.04137 | 0.18128 |
|  | 51~70 | 7 | 1.6846 | 0.76087 | 0.28758 |
| R7 | 41~50 | 33 | 1.6247 | 1.00503 | 0.17495 |
|  | 51~70 | 7 | 0.9924 | 0.55295 | 0.209 |
| R8 | 41~50 | 33 | 1.3639 | 0.82179 | 0.14306 |
|  | 51~70 | 7 | 0.7957 | 0.47613 | 0.17996 |
| R9 | 41~50 | 33 | 0.9104 | 0.53534 | 0.09319 |
|  | 51~70 | 7 | 0.5861 | 0.30761 | 0.11627 |

| **Group "41~50 age" & Group "51~70 age"** | | | | | | | | | | |
| --- | --- | --- | --- | --- | --- | --- | --- | --- | --- | --- |
|  |  | Levene's Test for Equality of Variances | | t-test for Equality of Means | |  |  |  |  |  |
|  |  | F | Sig. | t | df | Sig. (2-tailed) | Mean Difference | Std.Error Difference | 95% Confidence interval of the Difference | |
|  |  |  |  |  |  |  |  |  | Lower | Upper |
| L1 | Equal variances assumed | 0.787 | 0.381 | 0.357 | 38 | 0.723 | 0.07657 | 0.21433 | -0.35733 | 0.51046 |
|  | Equal variances not assumed |  |  | 0.501 | 14.75 | 0.624 | 0.07657 | 0.15282 | -0.24964 | 0.40277 |
| L2 | Equal variances assumed | 1.223 | 0.276 | 0.687 | 38 | 0.496 | 0.31709 | 0.46127 | -0.61669 | 1.25087 |
|  | Equal variances not assumed |  |  | 0.763 | 9.833 | 0.464 | 0.31709 | 0.41577 | -0.61143 | 1.24561 |
| L3 | Equal variances assumed | 1.151 | 0.29 | 0.013 | 38 | 0.99 | 0.00729 | 0.57178 | -1.15021 | 1.16478 |
|  | Equal variances not assumed |  |  | 0.011 | 7.543 | 0.992 | 0.00729 | 0.68501 | -1.58918 | 1.60376 |
| L4 | Equal variances assumed | 0.988 | 0.327 | 1.422 | 38 | 0.163 | 0.64088 | 0.45063 | -0.27137 | 1.55312 |
|  | Equal variances not assumed |  |  | 1.548 | 9.601 | 0.154 | 0.64088 | 0.41402 | -0.28684 | 1.5686 |
| L5 | Equal variances assumed | 0.313 | 0.579 | 1.75 | 38 | 0.088 | 0.78127 | 0.44636 | -0.12233 | 1.68487 |
|  | Equal variances not assumed |  |  | 1.912 | 9.645 | 0.086 | 0.78127 | 0.40858 | -0.13367 | 1.69621 |
| L6 | Equal variances assumed | 1.729 | 0.196 | 1.838 | 38 | 0.074 | 0.87758 | 0.47735 | -0.08877 | 1.84393 |
|  | Equal variances not assumed |  |  | 2.434 | 13.051 | 0.03 | 0.87758 | 0.36055 | 0.09897 | 1.65619 |
| L7 | Equal variances assumed | 3.732 | 0.061 | 1.608 | 38 | 0.116 | 0.88337 | 0.54941 | -0.22886 | 1.99559 |
|  | Equal variances not assumed |  |  | 2.811 | 27.73 | 0.009 | 0.88337 | 0.31423 | 0.23942 | 1.52731 |
| L8 | Equal variances assumed | 2.985 | 0.092 | 1.913 | 38 | 0.063 | 0.74304 | 0.3884 | -0.04323 | 1.52932 |
|  | Equal variances not assumed |  |  | 3.233 | 24.846 | 0.003 | 0.74304 | 0.22981 | 0.2696 | 1.21649 |
| L9 | Equal variances assumed | 0.034 | 0.855 | 1.132 | 38 | 0.265 | 0.25081 | 0.2216 | -0.1978 | 0.69941 |
|  | Equal variances not assumed |  |  | 1.23 | 9.586 | 0.248 | 0.25081 | 0.20385 | -0.20607 | 0.70768 |
| R1 | Equal variances assumed | 1.594 | 0.214 | 0.151 | 38 | 0.881 | 0.03043 | 0.20205 | -0.3786 | 0.43947 |
|  | Equal variances not assumed |  |  | 0.195 | 12.519 | 0.848 | 0.03043 | 0.15595 | -0.3078 | 0.36867 |
| R2 | Equal variances assumed | 0.13 | 0.721 | -0.357 | 38 | 0.723 | -0.15068 | 0.42257 | -1.00612 | 0.70476 |
|  | Equal variances not assumed |  |  | -0.412 | 10.401 | 0.688 | -0.15068 | 0.36534 | -0.96048 | 0.65913 |
| R3 | Equal variances assumed | 0.693 | 0.41 | -0.886 | 38 | 0.381 | -0.41965 | 0.47383 | -1.37888 | 0.53957 |
|  | Equal variances not assumed |  |  | -0.781 | 7.838 | 0.458 | -0.41965 | 0.53743 | -1.66344 | 0.82414 |
| R4 | Equal variances assumed | 1.053 | 0.311 | 0.904 | 38 | 0.372 | 0.36743 | 0.40661 | -0.4557 | 1.19057 |
|  | Equal variances not assumed |  |  | 1.103 | 11.284 | 0.293 | 0.36743 | 0.33311 | -0.36349 | 1.09836 |
| R5 | Equal variances assumed | 0.009 | 0.923 | 0.441 | 38 | 0.661 | 0.184 | 0.41679 | -0.65974 | 1.02774 |
|  | Equal variances not assumed |  |  | 0.412 | 8.203 | 0.691 | 0.184 | 0.44655 | -0.84133 | 1.20933 |
| R6 | Equal variances assumed | 0.814 | 0.373 | 1.272 | 38 | 0.211 | 0.5307 | 0.41709 | -0.31365 | 1.37505 |
|  | Equal variances not assumed |  |  | 1.561 | 11.379 | 0.146 | 0.5307 | 0.33995 | -0.21449 | 1.2759 |
| R7 | Equal variances assumed | 1.376 | 0.248 | 1.603 | 38 | 0.117 | 0.63224 | 0.39453 | -0.16644 | 1.43091 |
|  | Equal variances not assumed |  |  | 2.32 | 15.892 | 0.034 | 0.63224 | 0.27256 | 0.05412 | 1.21035 |
| R8 | Equal variances assumed | 1.706 | 0.199 | 1.756 | 38 | 0.087 | 0.56819 | 0.32354 | -0.08677 | 1.22316 |
|  | Equal variances not assumed |  |  | 2.472 | 14.866 | 0.026 | 0.56819 | 0.22989 | 0.0778 | 1.05859 |
| R9 | Equal variances assumed | 0.682 | 0.414 | 1.539 | 38 | 0.132 | 0.32425 | 0.21066 | -0.1022 | 0.75071 |
|  | Equal variances not assumed |  |  | 2.176 | 15.023 | 0.046 | 0.32425 | 0.149 | 0.0067 | 0.6418 |

| **Group "18~30 age" & Group "41~50 age"** | | | |  |  |
| --- | --- | --- | --- | --- | --- |
|  | Age | N | Mean | Std. Deviation | Std. Error Mean |
| L1 | 18~30 | 219 | 1.1819 | 0.77218 | 0.05218 |
|  | 41~50 | 33 | 1.0884 | 0.54421 | 0.09473 |
| L2 | 18~30 | 219 | 2.3575 | 1.26894 | 0.08575 |
|  | 41~50 | 33 | 2.2211 | 1.13281 | 0.1972 |
| L3 | 18~30 | 219 | 3.1287 | 1.31405 | 0.08879 |
|  | 41~50 | 33 | 2.931 | 1.30134 | 0.22653 |
| L4 | 18~30 | 219 | 3.4248 | 1.19665 | 0.08086 |
|  | 41~50 | 33 | 3.2559 | 1.10271 | 0.19196 |
| L5 | 18~30 | 219 | 3.2539 | 1.08755 | 0.07349 |
|  | 41~50 | 33 | 3.2227 | 1.09303 | 0.19027 |
| L6 | 18~30 | 219 | 2.8186 | 0.9573 | 0.06469 |
|  | 41~50 | 33 | 2.9602 | 1.20404 | 0.2096 |
| L7 | 18~30 | 219 | 2.0096 | 0.93286 | 0.06304 |
|  | 41~50 | 33 | 2.2639 | 1.42156 | 0.24746 |
| L8 | 18~30 | 219 | 1.725 | 0.87824 | 0.05935 |
|  | 41~50 | 33 | 1.8278 | 1.0026 | 0.17453 |
| L9 | 18~30 | 219 | 1.2619 | 0.6078 | 0.04107 |
|  | 41~50 | 33 | 1.1591 | 0.54213 | 0.09437 |
| R1 | 18~30 | 219 | 0.9619 | 0.62187 | 0.04202 |
|  | 41~50 | 33 | 0.9316 | 0.50826 | 0.08848 |
| R2 | 18~30 | 219 | 2.3188 | 1.18186 | 0.07986 |
|  | 41~50 | 33 | 2.0802 | 1.04538 | 0.18198 |
| R3 | 18~30 | 219 | 3.1256 | 1.15962 | 0.07836 |
|  | 41~50 | 33 | 2.7913 | 1.0994 | 0.19138 |
| R4 | 18~30 | 219 | 3.077 | 1.02719 | 0.06941 |
|  | 41~50 | 33 | 2.8406 | 1.01444 | 0.17659 |
| R5 | 18~30 | 219 | 2.8792 | 1.07474 | 0.07262 |
|  | 41~50 | 33 | 2.5076 | 0.9839 | 0.17127 |
| R6 | 18~30 | 219 | 2.322 | 1.03267 | 0.06978 |
|  | 41~50 | 33 | 2.2153 | 1.04137 | 0.18128 |
| R7 | 18~30 | 219 | 1.7486 | 1.07372 | 0.07256 |
|  | 41~50 | 33 | 1.6247 | 1.00503 | 0.17495 |
| R8 | 18~30 | 219 | 1.412 | 0.96939 | 0.06551 |
|  | 41~50 | 33 | 1.3639 | 0.82179 | 0.14306 |
| R9 | 18~30 | 219 | 0.9463 | 0.6049 | 0.04088 |
|  | 41~50 | 33 | 0.9104 | 0.53534 | 0.09319 |

| **Group "18~30 age" & Group "41~50 age"** | | | | | | | | | | |
| --- | --- | --- | --- | --- | --- | --- | --- | --- | --- | --- |
|  |  | Levene's Test for Equality of Variances | | t-test for Equality of Means | |  |  |  |  |  |
|  |  | F | Sig. | t | df | Sig. (2-tailed) | Mean Difference | Std.Error Difference | 95% Confidence interval of the Difference | |
|  |  |  |  |  |  |  |  |  | Lower | Upper |
| L1 | Equal variances assumed | 2.469 | 0.117 | 0.67 | 250 | 0.503 | 0.09349 | 0.13947 | -0.1812 | 0.36817 |
|  | Equal variances not assumed |  |  | 0.864 | 53.636 | 0.391 | 0.09349 | 0.10815 | -0.12338 | 0.31036 |
| L2 | Equal variances assumed | 0.35 | 0.555 | 0.583 | 250 | 0.56 | 0.13642 | 0.23385 | -0.32415 | 0.597 |
|  | Equal variances not assumed |  |  | 0.634 | 45.009 | 0.529 | 0.13642 | 0.21503 | -0.29667 | 0.56952 |
| L3 | Equal variances assumed | 0.076 | 0.783 | 0.807 | 250 | 0.421 | 0.1977 | 0.24507 | -0.28497 | 0.68037 |
|  | Equal variances not assumed |  |  | 0.813 | 42.441 | 0.421 | 0.1977 | 0.24331 | -0.29317 | 0.68858 |
| L4 | Equal variances assumed | 0.109 | 0.741 | 0.764 | 250 | 0.446 | 0.16896 | 0.22129 | -0.26687 | 0.60478 |
|  | Equal variances not assumed |  |  | 0.811 | 44.161 | 0.422 | 0.16896 | 0.20829 | -0.25079 | 0.5887 |
| L5 | Equal variances assumed | 0.015 | 0.904 | 0.154 | 250 | 0.878 | 0.03122 | 0.20321 | -0.36901 | 0.43145 |
|  | Equal variances not assumed |  |  | 0.153 | 42.122 | 0.879 | 0.03122 | 0.20397 | -0.38038 | 0.44282 |
| L6 | Equal variances assumed | 3.344 | 0.069 | -0.764 | 250 | 0.446 | -0.14152 | 0.1853 | -0.50646 | 0.22343 |
|  | Equal variances not assumed |  |  | -0.645 | 38.336 | 0.523 | -0.14152 | 0.21935 | -0.58544 | 0.30241 |
| L7 | Equal variances assumed | 9.978 | 0.002 | -1.35 | 250 | 0.178 | -0.25432 | 0.18836 | -0.6253 | 0.11665 |
|  | Equal variances not assumed |  |  | -0.996 | 36.265 | 0.326 | -0.25432 | 0.25536 | -0.77209 | 0.26345 |
| L8 | Equal variances assumed | 1.229 | 0.269 | -0.615 | 250 | 0.539 | -0.10277 | 0.16715 | -0.43197 | 0.22643 |
|  | Equal variances not assumed |  |  | -0.557 | 39.75 | 0.58 | -0.10277 | 0.18434 | -0.47541 | 0.26988 |
| L9 | Equal variances assumed | 0.296 | 0.587 | 0.918 | 250 | 0.36 | 0.10277 | 0.112 | -0.11781 | 0.32336 |
|  | Equal variances not assumed |  |  | 0.999 | 45.032 | 0.323 | 0.10277 | 0.10292 | -0.10452 | 0.31006 |
| R1 | Equal variances assumed | 0.474 | 0.492 | 0.267 | 250 | 0.79 | 0.03033 | 0.11363 | -0.19346 | 0.25412 |
|  | Equal variances not assumed |  |  | 0.31 | 47.709 | 0.758 | 0.03033 | 0.09795 | -0.16664 | 0.2273 |
| R2 | Equal variances assumed | 0.723 | 0.396 | 1.097 | 250 | 0.274 | 0.2386 | 0.2176 | -0.18995 | 0.66716 |
|  | Equal variances not assumed |  |  | 1.201 | 45.267 | 0.236 | 0.2386 | 0.19873 | -0.1616 | 0.6388 |
| R3 | Equal variances assumed | 0.524 | 0.47 | 1.554 | 250 | 0.122 | 0.33425 | 0.21513 | -0.08946 | 0.75795 |
|  | Equal variances not assumed |  |  | 1.616 | 43.449 | 0.113 | 0.33425 | 0.2068 | -0.08269 | 0.75118 |
| R4 | Equal variances assumed | 0.002 | 0.964 | 1.235 | 250 | 0.218 | 0.23646 | 0.19151 | -0.14072 | 0.61363 |
|  | Equal variances not assumed |  |  | 1.246 | 42.503 | 0.22 | 0.23646 | 0.18974 | -0.14633 | 0.61924 |
| R5 | Equal variances assumed | 0.899 | 0.344 | 1.871 | 250 | 0.062 | 0.37167 | 0.1986 | -0.01947 | 0.76281 |
|  | Equal variances not assumed |  |  | 1.998 | 44.331 | 0.052 | 0.37167 | 0.18604 | -0.00318 | 0.74653 |
| R6 | Equal variances assumed | 0.655 | 0.419 | 0.553 | 250 | 0.581 | 0.10672 | 0.19304 | -0.27348 | 0.48692 |
|  | Equal variances not assumed |  |  | 0.549 | 42.05 | 0.586 | 0.10672 | 0.19425 | -0.28527 | 0.49871 |
| R7 | Equal variances assumed | 0.468 | 0.495 | 0.623 | 250 | 0.534 | 0.12392 | 0.1989 | -0.26782 | 0.51566 |
|  | Equal variances not assumed |  |  | 0.654 | 43.764 | 0.516 | 0.12392 | 0.1894 | -0.25786 | 0.50569 |
| R8 | Equal variances assumed | 0.542 | 0.462 | 0.271 | 250 | 0.787 | 0.0481 | 0.17773 | -0.30193 | 0.39814 |
|  | Equal variances not assumed |  |  | 0.306 | 46.525 | 0.761 | 0.0481 | 0.15734 | -0.26851 | 0.36472 |
| R9 | Equal variances assumed | 0.764 | 0.383 | 0.322 | 250 | 0.747 | 0.03591 | 0.11138 | -0.18344 | 0.25527 |
|  | Equal variances not assumed |  |  | 0.353 | 45.251 | 0.726 | 0.03591 | 0.10176 | -0.16901 | 0.24084 |

| **Group "18~30 age" & Group "51~70 age"** | | | |  |  |
| --- | --- | --- | --- | --- | --- |
|  | Age | N | Mean | Std. Deviation | Std. Error Mean |
| L1 | 18~30 | 219 | 1.1819 | 0.77218 | 0.05218 |
|  | 51~70 | 7 | 1.0119 | 0.31726 | 0.11991 |
| L2 | 18~30 | 219 | 2.3575 | 1.26894 | 0.08575 |
|  | 51~70 | 7 | 1.904 | 0.96841 | 0.36603 |
| L3 | 18~30 | 219 | 3.1287 | 1.31405 | 0.08879 |
|  | 51~70 | 7 | 2.9237 | 1.7104 | 0.64647 |
| L4 | 18~30 | 219 | 3.4248 | 1.19665 | 0.08086 |
|  | 51~70 | 7 | 2.615 | 0.97054 | 0.36683 |
| L5 | 18~30 | 219 | 3.2539 | 1.08755 | 0.07349 |
|  | 51~70 | 7 | 2.4414 | 0.95663 | 0.36157 |
| L6 | 18~30 | 219 | 2.8186 | 0.9573 | 0.06469 |
|  | 51~70 | 7 | 2.0826 | 0.77619 | 0.29337 |
| L7 | 18~30 | 219 | 2.0096 | 0.93286 | 0.06304 |
|  | 51~70 | 7 | 1.3806 | 0.51235 | 0.19365 |
| L8 | 18~30 | 219 | 1.725 | 0.87824 | 0.05935 |
|  | 51~70 | 7 | 1.0847 | 0.39554 | 0.1495 |
| L9 | 18~30 | 219 | 1.2619 | 0.6078 | 0.04107 |
|  | 51~70 | 7 | 0.9083 | 0.47806 | 0.18069 |
| R1 | 18~30 | 219 | 0.9619 | 0.62187 | 0.04202 |
|  | 51~70 | 7 | 0.9011 | 0.33978 | 0.12842 |
| R2 | 18~30 | 219 | 2.3188 | 1.18186 | 0.07986 |
|  | 51~70 | 7 | 2.2309 | 0.83816 | 0.3168 |
| R3 | 18~30 | 219 | 3.1256 | 1.15962 | 0.07836 |
|  | 51~70 | 7 | 3.211 | 1.3287 | 0.5022 |
| R4 | 18~30 | 219 | 3.077 | 1.02719 | 0.06941 |
|  | 51~70 | 7 | 2.4731 | 0.74729 | 0.28245 |
| R5 | 18~30 | 219 | 2.8792 | 1.07474 | 0.07262 |
|  | 51~70 | 7 | 2.3236 | 1.09109 | 0.41239 |
| R6 | 18~30 | 219 | 2.322 | 1.03267 | 0.06978 |
|  | 51~70 | 7 | 1.6846 | 0.76087 | 0.28758 |
| R7 | 18~30 | 219 | 1.7486 | 1.07372 | 0.07256 |
|  | 51~70 | 7 | 0.9924 | 0.55295 | 0.209 |
| R8 | 18~30 | 219 | 1.412 | 0.96939 | 0.06551 |
|  | 51~70 | 7 | 0.7957 | 0.47613 | 0.17996 |
| R9 | 18~30 | 219 | 0.9463 | 0.6049 | 0.04088 |
|  | 51~70 | 7 | 0.5861 | 0.30761 | 0.11627 |

| **Group "18~30 age" & Group "51~70 age"** | | | | | | | | | | |
| --- | --- | --- | --- | --- | --- | --- | --- | --- | --- | --- |
|  |  | Levene's Test for Equality of Variances | | t-test for Equality of Means | |  |  |  |  |  |
|  |  | F | Sig. | t | df | Sig. (2-tailed) | Mean Difference | Std.Error Difference | 95% Confidence interval of the Difference | |
|  |  |  |  |  |  |  |  |  | Lower | Upper |
| L1 | Equal variances assumed | 1.89 | 0.171 | 0.58 | 224 | 0.562 | 0.17005 | 0.29316 | -0.40766 | 0.74777 |
|  | Equal variances not assumed |  |  | 1.3 | 8.479 | 0.228 | 0.17005 | 0.13077 | -0.12857 | 0.46868 |
| L2 | Equal variances assumed | 1.653 | 0.2 | 0.936 | 224 | 0.35 | 0.45351 | 0.48449 | -0.50122 | 1.40825 |
|  | Equal variances not assumed |  |  | 1.206 | 6.676 | 0.269 | 0.45351 | 0.37593 | -0.44425 | 1.35128 |
| L3 | Equal variances assumed | 1.384 | 0.241 | 0.403 | 224 | 0.688 | 0.20499 | 0.50921 | -0.79846 | 1.20844 |
|  | Equal variances not assumed |  |  | 0.314 | 6.228 | 0.764 | 0.20499 | 0.65254 | -1.37763 | 1.78761 |
| L4 | Equal variances assumed | 1.229 | 0.269 | 1.771 | 224 | 0.078 | 0.80984 | 0.45735 | -0.09143 | 1.7111 |
|  | Equal variances not assumed |  |  | 2.156 | 6.597 | 0.07 | 0.80984 | 0.37564 | -0.08953 | 1.7092 |
| L5 | Equal variances assumed | 0.448 | 0.504 | 1.952 | 224 | 0.052 | 0.81249 | 0.41631 | -0.00789 | 1.63287 |
|  | Equal variances not assumed |  |  | 2.202 | 6.506 | 0.066 | 0.81249 | 0.36896 | -0.07359 | 1.69857 |
| L6 | Equal variances assumed | 0.51 | 0.476 | 2.012 | 224 | 0.045 | 0.73606 | 0.36587 | 0.01508 | 1.45705 |
|  | Equal variances not assumed |  |  | 2.45 | 6.597 | 0.046 | 0.73606 | 0.30042 | 0.0168 | 1.45533 |
| L7 | Equal variances assumed | 1.327 | 0.251 | 1.773 | 224 | 0.078 | 0.62905 | 0.35481 | -0.07015 | 1.32825 |
|  | Equal variances not assumed |  |  | 3.089 | 7.337 | 0.017 | 0.62905 | 0.20365 | 0.15193 | 1.10617 |
| L8 | Equal variances assumed | 1.681 | 0.196 | 1.919 | 224 | 0.056 | 0.64027 | 0.33359 | -0.01709 | 1.29764 |
|  | Equal variances not assumed |  |  | 3.981 | 8.034 | 0.004 | 0.64027 | 0.16085 | 0.26963 | 1.01092 |
| L9 | Equal variances assumed | 0.186 | 0.667 | 1.523 | 224 | 0.129 | 0.35358 | 0.23217 | -0.10395 | 0.8111 |
|  | Equal variances not assumed |  |  | 1.908 | 6.636 | 0.1 | 0.35358 | 0.1853 | -0.08951 | 0.79667 |
| R1 | Equal variances assumed | 1.636 | 0.202 | 0.257 | 224 | 0.797 | 0.06077 | 0.23652 | -0.40532 | 0.52685 |
|  | Equal variances not assumed |  |  | 0.45 | 7.351 | 0.666 | 0.06077 | 0.13512 | -0.25568 | 0.37721 |
| R2 | Equal variances assumed | 0.544 | 0.461 | 0.195 | 224 | 0.846 | 0.08793 | 0.45075 | -0.80033 | 0.97619 |
|  | Equal variances not assumed |  |  | 0.269 | 6.786 | 0.796 | 0.08793 | 0.32671 | -0.68957 | 0.86543 |
| R3 | Equal variances assumed | 0.304 | 0.582 | -0.191 | 224 | 0.849 | -0.08541 | 0.44711 | -0.96648 | 0.79567 |
|  | Equal variances not assumed |  |  | -0.168 | 6.296 | 0.872 | -0.08541 | 0.50828 | -1.3151 | 1.14429 |
| R4 | Equal variances assumed | 0.986 | 0.322 | 1.541 | 224 | 0.125 | 0.60389 | 0.3919 | -0.1684 | 1.37618 |
|  | Equal variances not assumed |  |  | 2.076 | 6.746 | 0.078 | 0.60389 | 0.29085 | -0.08915 | 1.29693 |
| R5 | Equal variances assumed | 0.316 | 0.575 | 1.346 | 224 | 0.18 | 0.55568 | 0.41282 | -0.25784 | 1.36919 |
|  | Equal variances not assumed |  |  | 1.327 | 6.378 | 0.23 | 0.55568 | 0.41874 | -0.45441 | 1.56576 |
| R6 | Equal variances assumed | 2.377 | 0.125 | 1.618 | 224 | 0.107 | 0.63742 | 0.39407 | -0.13913 | 1.41397 |
|  | Equal variances not assumed |  |  | 2.154 | 6.727 | 0.07 | 0.63742 | 0.29593 | -0.06814 | 1.34298 |
| R7 | Equal variances assumed | 2.281 | 0.132 | 1.852 | 224 | 0.065 | 0.75616 | 0.40819 | -0.04822 | 1.56053 |
|  | Equal variances not assumed |  |  | 3.418 | 7.53 | 0.01 | 0.75616 | 0.22123 | 0.2404 | 1.27191 |
| R8 | Equal variances assumed | 2.053 | 0.153 | 1.673 | 224 | 0.096 | 0.6163 | 0.3684 | -0.10968 | 1.34228 |
|  | Equal variances not assumed |  |  | 3.218 | 7.692 | 0.013 | 0.6163 | 0.19151 | 0.17157 | 1.06103 |
| R9 | Equal variances assumed | 1.462 | 0.228 | 1.566 | 224 | 0.119 | 0.36016 | 0.22994 | -0.09295 | 0.81328 |
|  | Equal variances not assumed |  |  | 2.922 | 7.572 | 0.02 | 0.36016 | 0.12324 | 0.07315 | 0.64718 |

| **Group "31~40 age" & Group "51~70 age"** | | | |  |  |
| --- | --- | --- | --- | --- | --- |
|  | Age | N | Mean | Std. Deviation | Std. Error Mean |
| L1 | 31~40 | 141 | 1.0858 | 0.75747 | 0.06379 |
|  | 51~70 | 7 | 1.0119 | 0.31726 | 0.11991 |
| L2 | 31~40 | 141 | 2.2887 | 1.23796 | 0.10426 |
|  | 51~70 | 7 | 1.904 | 0.96841 | 0.36603 |
| L3 | 31~40 | 141 | 2.9304 | 1.18397 | 0.09971 |
|  | 51~70 | 7 | 2.9237 | 1.7104 | 0.64647 |
| L4 | 31~40 | 141 | 3.2429 | 1.05469 | 0.08882 |
|  | 51~70 | 7 | 2.615 | 0.97054 | 0.36683 |
| L5 | 31~40 | 141 | 3.1368 | 1.10835 | 0.09334 |
|  | 51~70 | 7 | 2.4414 | 0.95663 | 0.36157 |
| L6 | 31~40 | 141 | 2.7231 | 0.94422 | 0.07952 |
|  | 51~70 | 7 | 2.0826 | 0.77619 | 0.29337 |
| L7 | 31~40 | 141 | 2.1211 | 1.0674 | 0.08989 |
|  | 51~70 | 7 | 1.3806 | 0.51235 | 0.19365 |
| L8 | 31~40 | 141 | 1.8998 | 0.97717 | 0.08229 |
|  | 51~70 | 7 | 1.0847 | 0.39554 | 0.1495 |
| L9 | 31~40 | 141 | 1.452 | 0.78096 | 0.06577 |
|  | 51~70 | 7 | 0.9083 | 0.47806 | 0.18069 |
| R1 | 31~40 | 141 | 0.9125 | 0.54636 | 0.04601 |
|  | 51~70 | 7 | 0.9011 | 0.33978 | 0.12842 |
| R2 | 31~40 | 141 | 2.1405 | 1.0401 | 0.08759 |
|  | 51~70 | 7 | 2.2309 | 0.83816 | 0.3168 |
| R3 | 31~40 | 141 | 3.0118 | 1.18657 | 0.09993 |
|  | 51~70 | 7 | 3.211 | 1.3287 | 0.5022 |
| R4 | 31~40 | 141 | 3.165 | 1.25953 | 0.10607 |
|  | 51~70 | 7 | 2.4731 | 0.74729 | 0.28245 |
| R5 | 31~40 | 141 | 2.8917 | 1.21212 | 0.10208 |
|  | 51~70 | 7 | 2.3236 | 1.09109 | 0.41239 |
| R6 | 31~40 | 141 | 2.3555 | 1.16763 | 0.09833 |
|  | 51~70 | 7 | 1.6846 | 0.76087 | 0.28758 |
| R7 | 31~40 | 141 | 1.8614 | 1.27211 | 0.10713 |
|  | 51~70 | 7 | 0.9924 | 0.55295 | 0.209 |
| R8 | 31~40 | 141 | 1.4552 | 1.00544 | 0.08467 |
|  | 51~70 | 7 | 0.7957 | 0.47613 | 0.17996 |
| R9 | 31~40 | 141 | 0.9578 | 0.63681 | 0.05363 |
|  | 51~70 | 7 | 0.5861 | 0.30761 | 0.11627 |

| **Group "31~40 age" & Group "51~70 age"** | | | | | | | | | | |
| --- | --- | --- | --- | --- | --- | --- | --- | --- | --- | --- |
|  |  | Levene's Test for Equality of Variances | | t-test for Equality of Means | |  |  |  |  |  |
|  |  | F | Sig. | t | df | Sig. (2-tailed) | Mean Difference | Std.Error Difference | 95% Confidence interval of the Difference | |
|  |  |  |  |  |  |  |  |  | Lower | Upper |
| L1 | Equal variances assumed | 1.077 | 0.301 | 0.256 | 146 | 0.798 | 0.0739 | 0.2883 | -0.49589 | 0.64369 |
|  | Equal variances not assumed |  |  | 0.544 | 9.843 | 0.598 | 0.0739 | 0.13582 | -0.22939 | 0.37719 |
| L2 | Equal variances assumed | 0.913 | 0.341 | 0.809 | 146 | 0.42 | 0.38467 | 0.47554 | -0.55517 | 1.3245 |
|  | Equal variances not assumed |  |  | 1.011 | 7.011 | 0.346 | 0.38467 | 0.38058 | -0.51498 | 1.28432 |
| L3 | Equal variances assumed | 2.645 | 0.106 | 0.014 | 146 | 0.989 | 0.00672 | 0.4686 | -0.9194 | 0.93283 |
|  | Equal variances not assumed |  |  | 0.01 | 6.289 | 0.992 | 0.00672 | 0.65411 | -1.5762 | 1.58964 |
| L4 | Equal variances assumed | 1.339 | 0.249 | 1.542 | 146 | 0.125 | 0.62793 | 0.40712 | -0.17668 | 1.43255 |
|  | Equal variances not assumed |  |  | 1.664 | 6.723 | 0.142 | 0.62793 | 0.37743 | -0.27205 | 1.52792 |
| L5 | Equal variances assumed | 0.506 | 0.478 | 1.629 | 146 | 0.106 | 0.69537 | 0.42693 | -0.14839 | 1.53914 |
|  | Equal variances not assumed |  |  | 1.862 | 6.825 | 0.106 | 0.69537 | 0.37342 | -0.19224 | 1.58299 |
| L6 | Equal variances assumed | 0.682 | 0.41 | 1.764 | 146 | 0.08 | 0.64057 | 0.36319 | -0.07721 | 1.35835 |
|  | Equal variances not assumed |  |  | 2.107 | 6.912 | 0.074 | 0.64057 | 0.30396 | -0.08002 | 1.36116 |
| L7 | Equal variances assumed | 2.334 | 0.129 | 1.821 | 146 | 0.071 | 0.74055 | 0.40674 | -0.06332 | 1.54441 |
|  | Equal variances not assumed |  |  | 3.469 | 8.847 | 0.007 | 0.74055 | 0.2135 | 0.2563 | 1.22479 |
| L8 | Equal variances assumed | 2.681 | 0.104 | 2.192 | 146 | 0.03 | 0.81508 | 0.37183 | 0.08021 | 1.54995 |
|  | Equal variances not assumed |  |  | 4.776 | 10.147 | 0.001 | 0.81508 | 0.17065 | 0.43559 | 1.19458 |
| L9 | Equal variances assumed | 0.457 | 0.5 | 1.821 | 146 | 0.071 | 0.54368 | 0.2985 | -0.04626 | 1.13363 |
|  | Equal variances not assumed |  |  | 2.827 | 7.689 | 0.023 | 0.54368 | 0.19229 | 0.09713 | 0.99023 |
| R1 | Equal variances assumed | 2.138 | 0.146 | 0.055 | 146 | 0.957 | 0.0114 | 0.20888 | -0.40143 | 0.42422 |
|  | Equal variances not assumed |  |  | 0.084 | 7.634 | 0.936 | 0.0114 | 0.13642 | -0.30583 | 0.32862 |
| R2 | Equal variances assumed | 0.237 | 0.627 | -0.226 | 146 | 0.822 | -0.09033 | 0.39985 | -0.88057 | 0.6999 |
|  | Equal variances not assumed |  |  | -0.275 | 6.951 | 0.791 | -0.09033 | 0.32868 | -0.86866 | 0.68799 |
| R3 | Equal variances assumed | 0.203 | 0.653 | -0.431 | 146 | 0.667 | -0.19923 | 0.46187 | -1.11204 | 0.71359 |
|  | Equal variances not assumed |  |  | -0.389 | 6.484 | 0.71 | -0.19923 | 0.51205 | -1.42983 | 1.03138 |
| R4 | Equal variances assumed | 2.238 | 0.137 | 1.438 | 146 | 0.153 | 0.69183 | 0.48119 | -0.25918 | 1.64283 |
|  | Equal variances not assumed |  |  | 2.293 | 7.805 | 0.052 | 0.69183 | 0.30171 | -0.00695 | 1.39061 |
| R5 | Equal variances assumed | 0.44 | 0.508 | 1.215 | 146 | 0.226 | 0.56813 | 0.46754 | -0.35589 | 1.49215 |
|  | Equal variances not assumed |  |  | 1.337 | 6.757 | 0.224 | 0.56813 | 0.42484 | -0.44384 | 1.5801 |
| R6 | Equal variances assumed | 1.908 | 0.169 | 1.502 | 146 | 0.135 | 0.67089 | 0.44677 | -0.21208 | 1.55386 |
|  | Equal variances not assumed |  |  | 2.207 | 7.481 | 0.061 | 0.67089 | 0.30393 | -0.03853 | 1.38031 |
| R7 | Equal variances assumed | 2.255 | 0.135 | 1.794 | 146 | 0.075 | 0.869 | 0.48433 | -0.08819 | 1.8262 |
|  | Equal variances not assumed |  |  | 3.7 | 9.539 | 0.004 | 0.869 | 0.23485 | 0.34227 | 1.39573 |
| R8 | Equal variances assumed | 1.992 | 0.16 | 1.722 | 146 | 0.087 | 0.6595 | 0.38308 | -0.0976 | 1.4166 |
|  | Equal variances not assumed |  |  | 3.316 | 8.932 | 0.009 | 0.6595 | 0.19889 | 0.20906 | 1.10993 |
| R9 | Equal variances assumed | 1.367 | 0.244 | 1.531 | 146 | 0.128 | 0.37165 | 0.24268 | -0.10797 | 0.85127 |
|  | Equal variances not assumed |  |  | 2.903 | 8.808 | 0.018 | 0.37165 | 0.12804 | 0.08104 | 0.66226 |
