# Supplementary material for: Quantitative analysis of the labia minora morphology in 400 Chinese women: A new method for assessing the shape of the labia minora
Source: Front Surg. 2023 Jan 6;9:961247. doi: 10.3389/fsurg.2022.961247 (PMC9852508; doi:10.3389/fsurg.2022.961247)
Supplement: Supplementary file 2 [file Table2.docx]

**Supplementary file 2. Group statistics and independent samples t-tests based on the number of births.**

| **Group "nulliparous women" & Group "primiparous women"** | | |  |  |  |
| --- | --- | --- | --- | --- | --- |
|  |  | N | Mean | Std. Deviation | Std. Error Mean |
| L1 | 0 birth | 243 | 1.1667 | 0.76335 | 0.04897 |
|  | 1 birth | 99 | 1.1252 | 0.78577 | 0.07897 |
| L2 | 0 birth | 243 | 2.3191 | 1.2237 | 0.0785 |
|  | 1 birth | 99 | 2.2775 | 1.25709 | 0.12634 |
| L3 | 0 birth | 243 | 3.0589 | 1.29831 | 0.08329 |
|  | 1 birth | 99 | 3.0548 | 1.20141 | 0.12075 |
| L4 | 0 birth | 243 | 3.3374 | 1.16273 | 0.07459 |
|  | 1 birth | 99 | 3.4421 | 1.15692 | 0.11628 |
| L5 | 0 birth | 243 | 3.1865 | 1.1143 | 0.07148 |
|  | 1 birth | 99 | 3.3136 | 1.1352 | 0.11409 |
| L6 | 0 birth | 243 | 2.7734 | 0.94013 | 0.06031 |
|  | 1 birth | 99 | 2.8539 | 1.05186 | 0.10572 |
| L7 | 0 birth | 243 | 2.0352 | 0.95307 | 0.06114 |
|  | 1 birth | 99 | 2.0697 | 1.05963 | 0.1065 |
| L8 | 0 birth | 243 | 1.7767 | 0.93823 | 0.06019 |
|  | 1 birth | 99 | 1.7257 | 0.80181 | 0.08058 |
| L9 | 0 birth | 243 | 1.2905 | 0.66412 | 0.0426 |
|  | 1 birth | 99 | 1.3645 | 0.7417 | 0.07454 |
| R1 | 0 birth | 243 | 0.9661 | 0.62714 | 0.04023 |
|  | 1 birth | 99 | 0.9369 | 0.54312 | 0.05459 |
| R2 | 0 birth | 243 | 2.2727 | 1.14037 | 0.07315 |
|  | 1 birth | 99 | 2.1383 | 1.05053 | 0.10558 |
| R3 | 0 birth | 243 | 3.1119 | 1.19372 | 0.07658 |
|  | 1 birth | 99 | 3.0456 | 1.13137 | 0.11371 |
| R4 | 0 birth | 243 | 3.1011 | 1.12261 | 0.07202 |
|  | 1 birth | 99 | 3.1783 | 1.18498 | 0.11909 |
| R5 | 0 birth | 243 | 2.8828 | 1.13499 | 0.07281 |
|  | 1 birth | 99 | 2.8734 | 1.16056 | 0.11664 |
| R6 | 0 birth | 243 | 2.3049 | 1.05987 | 0.06799 |
|  | 1 birth | 99 | 2.413 | 1.22191 | 0.12281 |
| R7 | 0 birth | 243 | 1.7103 | 1.08491 | 0.0696 |
|  | 1 birth | 99 | 1.8979 | 1.31319 | 0.13198 |
| R8 | 0 birth | 243 | 1.3713 | 0.9173 | 0.05884 |
|  | 1 birth | 99 | 1.4015 | 0.91836 | 0.0923 |
| R9 | 0 birth | 243 | 0.9399 | 0.60264 | 0.03866 |
|  | 1 birth | 99 | 0.9278 | 0.5709 | 0.05738 |

| **Group "nulliparous women" & Group "primiparous women"** | | | | | | | | | | |
| --- | --- | --- | --- | --- | --- | --- | --- | --- | --- | --- |
|  |  | Levene's Test for Equality of Variances | | t-test for Equality of Means | | |  |  |  |  |
|  |  | F | Sig. | t | df | Sig. (2-tailed) | Mean Difference | Std.Error Difference | 95% Confidence interval of the Difference | |
|  |  |  |  |  |  |  |  |  | Lower | Upper |
| L1 | Equal variances assumed | 1.329 | 0.25 | 0.452 | 340 | 0.651 | 0.04152 | 0.09179 | -0.13904 | 0.22207 |
|  | Equal variances not assumed |  |  | 0.447 | 177.237 | 0.656 | 0.04152 | 0.09292 | -0.14186 | 0.22489 |
| L2 | Equal variances assumed | 0.175 | 0.676 | 0.283 | 340 | 0.778 | 0.04159 | 0.14706 | -0.24768 | 0.33085 |
|  | Equal variances not assumed |  |  | 0.28 | 177.556 | 0.78 | 0.04159 | 0.14874 | -0.25194 | 0.33512 |
| L3 | Equal variances assumed | 0.026 | 0.871 | 0.027 | 340 | 0.978 | 0.00413 | 0.15156 | -0.29398 | 0.30224 |
|  | Equal variances not assumed |  |  | 0.028 | 195.514 | 0.978 | 0.00413 | 0.14668 | -0.28516 | 0.29342 |
| L4 | Equal variances assumed | 0.52 | 0.471 | -0.756 | 340 | 0.45 | -0.1047 | 0.13844 | -0.377 | 0.1676 |
|  | Equal variances not assumed |  |  | -0.758 | 182.721 | 0.449 | -0.1047 | 0.13814 | -0.37726 | 0.16786 |
| L5 | Equal variances assumed | 0.122 | 0.727 | -0.952 | 340 | 0.342 | -0.12713 | 0.13358 | -0.38989 | 0.13562 |
|  | Equal variances not assumed |  |  | -0.944 | 178.878 | 0.346 | -0.12713 | 0.13464 | -0.39281 | 0.13854 |
| L6 | Equal variances assumed | 0.134 | 0.715 | -0.694 | 340 | 0.488 | -0.08054 | 0.11609 | -0.30888 | 0.14781 |
|  | Equal variances not assumed |  |  | -0.662 | 165.087 | 0.509 | -0.08054 | 0.12171 | -0.32085 | 0.15977 |
| L7 | Equal variances assumed | 0 | 0.985 | -0.294 | 340 | 0.769 | -0.0345 | 0.11744 | -0.2655 | 0.1965 |
|  | Equal variances not assumed |  |  | -0.281 | 165.945 | 0.779 | -0.0345 | 0.1228 | -0.27695 | 0.20795 |
| L8 | Equal variances assumed | 1.783 | 0.183 | 0.475 | 340 | 0.635 | 0.05102 | 0.10743 | -0.1603 | 0.26233 |
|  | Equal variances not assumed |  |  | 0.507 | 211.217 | 0.613 | 0.05102 | 0.10058 | -0.14725 | 0.24929 |
| L9 | Equal variances assumed | 0.013 | 0.909 | -0.903 | 340 | 0.367 | -0.07404 | 0.08196 | -0.23525 | 0.08717 |
|  | Equal variances not assumed |  |  | -0.862 | 165.334 | 0.39 | -0.07404 | 0.08586 | -0.24356 | 0.09548 |
| R1 | Equal variances assumed | 0.67 | 0.414 | 0.406 | 340 | 0.685 | 0.02928 | 0.07203 | -0.1124 | 0.17096 |
|  | Equal variances not assumed |  |  | 0.432 | 208.478 | 0.666 | 0.02928 | 0.06781 | -0.1044 | 0.16296 |
| R2 | Equal variances assumed | 1.279 | 0.259 | 1.011 | 340 | 0.313 | 0.13445 | 0.13297 | -0.12709 | 0.396 |
|  | Equal variances not assumed |  |  | 1.047 | 196.354 | 0.297 | 0.13445 | 0.12845 | -0.11887 | 0.38777 |
| R3 | Equal variances assumed | 0.034 | 0.853 | 0.473 | 340 | 0.637 | 0.06626 | 0.14023 | -0.20956 | 0.34208 |
|  | Equal variances not assumed |  |  | 0.483 | 191.132 | 0.629 | 0.06626 | 0.13709 | -0.20414 | 0.33666 |
| R4 | Equal variances assumed | 0.228 | 0.633 | -0.567 | 340 | 0.571 | -0.07716 | 0.13604 | -0.34474 | 0.19041 |
|  | Equal variances not assumed |  |  | -0.554 | 173.382 | 0.58 | -0.07716 | 0.13918 | -0.35186 | 0.19753 |
| R5 | Equal variances assumed | 0.025 | 0.873 | 0.069 | 340 | 0.945 | 0.00941 | 0.13621 | -0.25851 | 0.27734 |
|  | Equal variances not assumed |  |  | 0.068 | 178.29 | 0.946 | 0.00941 | 0.1375 | -0.26192 | 0.28075 |
| R6 | Equal variances assumed | 1.373 | 0.242 | -0.817 | 340 | 0.414 | -0.10809 | 0.13223 | -0.36818 | 0.152 |
|  | Equal variances not assumed |  |  | -0.77 | 161.154 | 0.442 | -0.10809 | 0.14037 | -0.3853 | 0.16911 |
| R7 | Equal variances assumed | 1.252 | 0.264 | -1.361 | 340 | 0.174 | -0.18755 | 0.13775 | -0.45851 | 0.08341 |
|  | Equal variances not assumed |  |  | -1.257 | 155.22 | 0.211 | -0.18755 | 0.14921 | -0.48229 | 0.10719 |
| R8 | Equal variances assumed | 0.047 | 0.829 | -0.277 | 340 | 0.782 | -0.03027 | 0.10941 | -0.24548 | 0.18493 |
|  | Equal variances not assumed |  |  | -0.277 | 181.701 | 0.782 | -0.03027 | 0.10946 | -0.24625 | 0.18571 |
| R9 | Equal variances assumed | 0.073 | 0.787 | 0.171 | 340 | 0.864 | 0.01213 | 0.07078 | -0.1271 | 0.15136 |
|  | Equal variances not assumed |  |  | 0.175 | 191.215 | 0.861 | 0.01213 | 0.06919 | -0.12433 | 0.1486 |

| **Group "primiparous women" & Group "multiparous women"** | | | | | |
| --- | --- | --- | --- | --- | --- |
|  |  | N | Mean | Std. Deviation | Std. Error Mean |
| L1 | 1 birth | 99 | 1.1252 | 0.78577 | 0.07897 |
|  | ≥2 births | 58 | 1.0351 | 0.57793 | 0.07589 |
| L2 | 1 birth | 99 | 2.2775 | 1.25709 | 0.12634 |
|  | ≥2 births | 58 | 2.3552 | 1.30288 | 0.17108 |
| L3 | 1 birth | 99 | 3.0548 | 1.20141 | 0.12075 |
|  | ≥2 births | 58 | 2.928 | 1.31197 | 0.17227 |
| L4 | 1 birth | 99 | 3.4421 | 1.15692 | 0.11628 |
|  | ≥2 births | 58 | 3.1257 | 1.00214 | 0.13159 |
| L5 | 1 birth | 99 | 3.3136 | 1.1352 | 0.11409 |
|  | ≥2 births | 58 | 3.0341 | 0.92811 | 0.12187 |
| L6 | 1 birth | 99 | 2.8539 | 1.05186 | 0.10572 |
|  | ≥2 births | 58 | 2.7076 | 0.99724 | 0.13094 |
| L7 | 1 birth | 99 | 2.0697 | 1.05963 | 0.1065 |
|  | ≥2 births | 58 | 2.1396 | 1.26189 | 0.16569 |
| L8 | 1 birth | 99 | 1.7257 | 0.80181 | 0.08058 |
|  | ≥2 births | 58 | 1.9131 | 1.05731 | 0.13883 |
| L9 | 1 birth | 99 | 1.3645 | 0.7417 | 0.07454 |
|  | ≥2 births | 58 | 1.3277 | 0.60517 | 0.07946 |
| R1 | 1 birth | 99 | 0.9369 | 0.54312 | 0.05459 |
|  | ≥2 births | 58 | 0.8423 | 0.42857 | 0.05627 |
| R2 | 1 birth | 99 | 2.1383 | 1.05053 | 0.10558 |
|  | ≥2 births | 58 | 2.2403 | 1.14398 | 0.15021 |
| R3 | 1 birth | 99 | 3.0456 | 1.13137 | 0.11371 |
|  | ≥2 births | 58 | 2.8631 | 1.10929 | 0.14566 |
| R4 | 1 birth | 99 | 3.1783 | 1.18498 | 0.11909 |
|  | ≥2 births | 58 | 2.8098 | 0.89834 | 0.11796 |
| R5 | 1 birth | 99 | 2.8734 | 1.16056 | 0.11664 |
|  | ≥2 births | 58 | 2.6262 | 0.98137 | 0.12886 |
| R6 | 1 birth | 99 | 2.413 | 1.22191 | 0.12281 |
|  | ≥2 births | 58 | 2.182 | 0.88563 | 0.11629 |
| R7 | 1 birth | 99 | 1.8979 | 1.31319 | 0.13198 |
|  | ≥2 births | 58 | 1.7665 | 1.04232 | 0.13686 |
| R8 | 1 birth | 99 | 1.4015 | 0.91836 | 0.0923 |
|  | ≥2 births | 58 | 1.604 | 1.21048 | 0.15894 |
| R9 | 1 birth | 99 | 0.9278 | 0.5709 | 0.05738 |
|  | ≥2 births | 58 | 0.9686 | 0.69264 | 0.09095 |

| **Group "primiparous women" & Group "multiparous women"** | | | | | | | | | | |
| --- | --- | --- | --- | --- | --- | --- | --- | --- | --- | --- |
|  |  | Levene's Test for Equality of Variances | | t-test for Equality of Means | | |  |  |  |  |
|  |  | F | Sig. | t | df | Sig. (2-tailed) | Mean Difference | Std.Error Difference | 95% Confidence interval of the Difference | |
|  |  |  |  |  |  |  |  |  | Lower | Upper |
| L1 | Equal variances assumed | 0.065 | 0.8 | 0.76 | 155 | 0.448 | 0.09002 | 0.11846 | -0.14398 | 0.32402 |
|  | Equal variances not assumed |  |  | 0.822 | 147.021 | 0.412 | 0.09002 | 0.10952 | -0.12642 | 0.30647 |
| L2 | Equal variances assumed | 0.083 | 0.774 | -0.369 | 155 | 0.713 | -0.07768 | 0.21068 | -0.49386 | 0.3385 |
|  | Equal variances not assumed |  |  | -0.365 | 116.052 | 0.716 | -0.07768 | 0.21267 | -0.4989 | 0.34354 |
| L3 | Equal variances assumed | 0.084 | 0.772 | 0.617 | 155 | 0.538 | 0.12677 | 0.20557 | -0.27931 | 0.53285 |
|  | Equal variances not assumed |  |  | 0.603 | 111.159 | 0.548 | 0.12677 | 0.21037 | -0.29009 | 0.54363 |
| L4 | Equal variances assumed | 2.881 | 0.092 | 1.735 | 155 | 0.085 | 0.31636 | 0.18231 | -0.04377 | 0.67649 |
|  | Equal variances not assumed |  |  | 1.802 | 133.444 | 0.074 | 0.31636 | 0.1756 | -0.03096 | 0.66368 |
| L5 | Equal variances assumed | 1.701 | 0.194 | 1.589 | 155 | 0.114 | 0.27954 | 0.17589 | -0.06792 | 0.627 |
|  | Equal variances not assumed |  |  | 1.675 | 138.722 | 0.096 | 0.27954 | 0.16694 | -0.05053 | 0.60961 |
| L6 | Equal variances assumed | 0.004 | 0.947 | 0.858 | 155 | 0.392 | 0.14637 | 0.17067 | -0.19076 | 0.4835 |
|  | Equal variances not assumed |  |  | 0.87 | 124.706 | 0.386 | 0.14637 | 0.16829 | -0.18671 | 0.47945 |
| L7 | Equal variances assumed | 2.988 | 0.086 | -0.371 | 155 | 0.711 | -0.06984 | 0.18821 | -0.44162 | 0.30194 |
|  | Equal variances not assumed |  |  | -0.355 | 103.544 | 0.724 | -0.06984 | 0.19697 | -0.46045 | 0.32077 |
| L8 | Equal variances assumed | 3.065 | 0.082 | -1.253 | 155 | 0.212 | -0.18739 | 0.14951 | -0.48273 | 0.10796 |
|  | Equal variances not assumed |  |  | -1.167 | 95.569 | 0.246 | -0.18739 | 0.16052 | -0.50604 | 0.13127 |
| L9 | Equal variances assumed | 0.032 | 0.858 | 0.321 | 155 | 0.749 | 0.03688 | 0.11486 | -0.19001 | 0.26377 |
|  | Equal variances not assumed |  |  | 0.338 | 138.9 | 0.736 | 0.03688 | 0.10895 | -0.17854 | 0.2523 |
| R1 | Equal variances assumed | 3.414 | 0.067 | 1.135 | 155 | 0.258 | 0.09459 | 0.08334 | -0.07004 | 0.25923 |
|  | Equal variances not assumed |  |  | 1.207 | 141.74 | 0.23 | 0.09459 | 0.0784 | -0.06039 | 0.24957 |
| R2 | Equal variances assumed | 1.25 | 0.265 | -0.568 | 155 | 0.571 | -0.10202 | 0.17955 | -0.4567 | 0.25265 |
|  | Equal variances not assumed |  |  | -0.556 | 111.417 | 0.58 | -0.10202 | 0.18361 | -0.46583 | 0.26179 |
| R3 | Equal variances assumed | 0.051 | 0.822 | 0.982 | 155 | 0.328 | 0.18245 | 0.18574 | -0.18447 | 0.54936 |
|  | Equal variances not assumed |  |  | 0.987 | 121.416 | 0.325 | 0.18245 | 0.18478 | -0.18337 | 0.54826 |
| R4 | Equal variances assumed | 3.151 | 0.078 | 2.047 | 155 | 0.042 | 0.36849 | 0.17997 | 0.01298 | 0.724 |
|  | Equal variances not assumed |  |  | 2.198 | 144.877 | 0.03 | 0.36849 | 0.16762 | 0.03718 | 0.69979 |
| R5 | Equal variances assumed | 1.852 | 0.175 | 1.361 | 155 | 0.175 | 0.24718 | 0.18157 | -0.11149 | 0.60586 |
|  | Equal variances not assumed |  |  | 1.422 | 135.688 | 0.157 | 0.24718 | 0.17381 | -0.09654 | 0.59091 |
| R6 | Equal variances assumed | 3.802 | 0.053 | 1.258 | 155 | 0.21 | 0.23099 | 0.18357 | -0.13163 | 0.59361 |
|  | Equal variances not assumed |  |  | 1.366 | 147.98 | 0.174 | 0.23099 | 0.16913 | -0.10323 | 0.56521 |
| R7 | Equal variances assumed | 1.214 | 0.272 | 0.651 | 155 | 0.516 | 0.13134 | 0.20183 | -0.26735 | 0.53004 |
|  | Equal variances not assumed |  |  | 0.691 | 141.255 | 0.491 | 0.13134 | 0.19013 | -0.24453 | 0.50722 |
| R8 | Equal variances assumed | 2.38 | 0.125 | -1.182 | 155 | 0.239 | -0.20244 | 0.17121 | -0.54065 | 0.13577 |
|  | Equal variances not assumed |  |  | -1.101 | 95.601 | 0.273 | -0.20244 | 0.1838 | -0.5673 | 0.16242 |
| R9 | Equal variances assumed | 0.004 | 0.947 | -0.399 | 155 | 0.69 | -0.04082 | 0.10227 | -0.24284 | 0.16119 |
|  | Equal variances not assumed |  |  | -0.38 | 102.005 | 0.705 | -0.04082 | 0.10754 | -0.25412 | 0.17247 |

| **Group "nulliparous women" & Group "multiparous women"** | | | | | |
| --- | --- | --- | --- | --- | --- |
|  |  | N | Mean | Std. Deviation | Std. Error Mean |
| L1 | 0 birth | 243 | 1.1667 | 0.76335 | 0.04897 |
|  | ≥2 births | 58 | 1.0351 | 0.57793 | 0.07589 |
| L2 | 0 birth | 243 | 2.3191 | 1.2237 | 0.0785 |
|  | ≥2 births | 58 | 2.3552 | 1.30288 | 0.17108 |
| L3 | 0 birth | 243 | 3.0589 | 1.29831 | 0.08329 |
|  | ≥2 births | 58 | 2.928 | 1.31197 | 0.17227 |
| L4 | 0 birth | 243 | 3.3374 | 1.16273 | 0.07459 |
|  | ≥2 births | 58 | 3.1257 | 1.00214 | 0.13159 |
| L5 | 0 birth | 243 | 3.1865 | 1.1143 | 0.07148 |
|  | ≥2 births | 58 | 3.0341 | 0.92811 | 0.12187 |
| L6 | 0 birth | 243 | 2.7734 | 0.94013 | 0.06031 |
|  | ≥2 births | 58 | 2.7076 | 0.99724 | 0.13094 |
| L7 | 0 birth | 243 | 2.0352 | 0.95307 | 0.06114 |
|  | ≥2 births | 58 | 2.1396 | 1.26189 | 0.16569 |
| L8 | 0 birth | 243 | 1.7767 | 0.93823 | 0.06019 |
|  | ≥2 births | 58 | 1.9131 | 1.05731 | 0.13883 |
| L9 | 0 birth | 243 | 1.2905 | 0.66412 | 0.0426 |
|  | ≥2 births | 58 | 1.3277 | 0.60517 | 0.07946 |
| R1 | 0 birth | 243 | 0.9661 | 0.62714 | 0.04023 |
|  | ≥2 births | 58 | 0.8423 | 0.42857 | 0.05627 |
| R2 | 0 birth | 243 | 2.2727 | 1.14037 | 0.07315 |
|  | ≥2 births | 58 | 2.2403 | 1.14398 | 0.15021 |
| R3 | 0 birth | 243 | 3.1119 | 1.19372 | 0.07658 |
|  | ≥2 births | 58 | 2.8631 | 1.10929 | 0.14566 |
| R4 | 0 birth | 243 | 3.1011 | 1.12261 | 0.07202 |
|  | ≥2 births | 58 | 2.8098 | 0.89834 | 0.11796 |
| R5 | 0 birth | 243 | 2.8828 | 1.13499 | 0.07281 |
|  | ≥2 births | 58 | 2.6262 | 0.98137 | 0.12886 |
| R6 | 0 birth | 243 | 2.3049 | 1.05987 | 0.06799 |
|  | ≥2 births | 58 | 2.182 | 0.88563 | 0.11629 |
| R7 | 0 birth | 243 | 1.7103 | 1.08491 | 0.0696 |
|  | ≥2 births | 58 | 1.7665 | 1.04232 | 0.13686 |
| R8 | 0 birth | 243 | 1.3713 | 0.9173 | 0.05884 |
|  | ≥2 births | 58 | 1.604 | 1.21048 | 0.15894 |
| R9 | 0 birth | 243 | 0.9399 | 0.60264 | 0.03866 |
|  | ≥2 births | 58 | 0.9686 | 0.69264 | 0.09095 |

| **Group "nulliparous women" & Group "multiparous women"** | | | | | | | | | | |
| --- | --- | --- | --- | --- | --- | --- | --- | --- | --- | --- |
|  |  | Levene's Test for Equality of Variances | | t-test for Equality of Means | | |  |  |  |  |
|  |  | F | Sig. | t | df | Sig. (2-tailed) | Mean Difference | Std.Error Difference | 95% Confidence interval of the Difference | |
|  |  |  |  |  |  |  |  |  | Lower | Upper |
| L1 | Equal variances assumed | 1.831 | 0.177 | 1.23 | 299 | 0.22 | 0.13154 | 0.10692 | -0.07887 | 0.34195 |
|  | Equal variances not assumed |  |  | 1.456 | 109.867 | 0.148 | 0.13154 | 0.09031 | -0.04744 | 0.31052 |
| L2 | Equal variances assumed | 0.445 | 0.505 | -0.199 | 299 | 0.842 | -0.03609 | 0.18109 | -0.39247 | 0.32028 |
|  | Equal variances not assumed |  |  | -0.192 | 82.667 | 0.848 | -0.03609 | 0.18823 | -0.41049 | 0.3383 |
| L3 | Equal variances assumed | 0.163 | 0.687 | 0.689 | 299 | 0.492 | 0.1309 | 0.19012 | -0.24324 | 0.50504 |
|  | Equal variances not assumed |  |  | 0.684 | 85.658 | 0.496 | 0.1309 | 0.19135 | -0.24951 | 0.51131 |
| L4 | Equal variances assumed | 1.261 | 0.262 | 1.277 | 299 | 0.202 | 0.21166 | 0.1657 | -0.11443 | 0.53776 |
|  | Equal variances not assumed |  |  | 1.399 | 97.152 | 0.165 | 0.21166 | 0.15126 | -0.08854 | 0.51186 |
| L5 | Equal variances assumed | 1.223 | 0.27 | 0.964 | 299 | 0.336 | 0.1524 | 0.15802 | -0.15856 | 0.46337 |
|  | Equal variances not assumed |  |  | 1.079 | 100.176 | 0.283 | 0.1524 | 0.14128 | -0.12789 | 0.4327 |
| L6 | Equal variances assumed | 0.052 | 0.821 | 0.474 | 299 | 0.636 | 0.06583 | 0.13902 | -0.20775 | 0.33941 |
|  | Equal variances not assumed |  |  | 0.457 | 82.869 | 0.649 | 0.06583 | 0.14416 | -0.22092 | 0.35257 |
| L7 | Equal variances assumed | 5.542 | 0.019 | -0.701 | 299 | 0.484 | -0.10434 | 0.14894 | -0.39745 | 0.18877 |
|  | Equal variances not assumed |  |  | -0.591 | 73.258 | 0.556 | -0.10434 | 0.17661 | -0.45631 | 0.24763 |
| L8 | Equal variances assumed | 0.704 | 0.402 | -0.97 | 299 | 0.333 | -0.13637 | 0.1406 | -0.41305 | 0.14032 |
|  | Equal variances not assumed |  |  | -0.901 | 79.776 | 0.37 | -0.13637 | 0.15132 | -0.43751 | 0.16478 |
| L9 | Equal variances assumed | 0.015 | 0.902 | -0.389 | 299 | 0.697 | -0.03716 | 0.09547 | -0.22504 | 0.15072 |
|  | Equal variances not assumed |  |  | -0.412 | 92.675 | 0.681 | -0.03716 | 0.09016 | -0.21621 | 0.14189 |
| R1 | Equal variances assumed | 5.339 | 0.022 | 1.426 | 299 | 0.155 | 0.12387 | 0.08687 | -0.04708 | 0.29482 |
|  | Equal variances not assumed |  |  | 1.791 | 122.614 | 0.076 | 0.12387 | 0.06918 | -0.01306 | 0.26081 |
| R2 | Equal variances assumed | 0.087 | 0.768 | 0.194 | 299 | 0.846 | 0.03243 | 0.16675 | -0.29573 | 0.36059 |
|  | Equal variances not assumed |  |  | 0.194 | 86.104 | 0.847 | 0.03243 | 0.16708 | -0.29971 | 0.36456 |
| R3 | Equal variances assumed | 0.143 | 0.705 | 1.445 | 299 | 0.15 | 0.24871 | 0.17217 | -0.0901 | 0.58752 |
|  | Equal variances not assumed |  |  | 1.511 | 91.223 | 0.134 | 0.24871 | 0.16456 | -0.07816 | 0.57557 |
| R4 | Equal variances assumed | 2.577 | 0.109 | 1.84 | 299 | 0.067 | 0.29132 | 0.15833 | -0.02026 | 0.60291 |
|  | Equal variances not assumed |  |  | 2.108 | 104.007 | 0.037 | 0.29132 | 0.1382 | 0.01726 | 0.56539 |
| R5 | Equal variances assumed | 3.047 | 0.082 | 1.586 | 299 | 0.114 | 0.2566 | 0.16183 | -0.06187 | 0.57506 |
|  | Equal variances not assumed |  |  | 1.734 | 96.88 | 0.086 | 0.2566 | 0.14801 | -0.03716 | 0.55035 |
| R6 | Equal variances assumed | 2.032 | 0.155 | 0.817 | 299 | 0.414 | 0.1229 | 0.15037 | -0.17301 | 0.41881 |
|  | Equal variances not assumed |  |  | 0.912 | 99.882 | 0.364 | 0.1229 | 0.13471 | -0.14436 | 0.39016 |
| R7 | Equal variances assumed | 0.253 | 0.615 | -0.357 | 299 | 0.721 | -0.05621 | 0.15738 | -0.36592 | 0.25351 |
|  | Equal variances not assumed |  |  | -0.366 | 88.89 | 0.715 | -0.05621 | 0.15354 | -0.3613 | 0.24888 |
| R8 | Equal variances assumed | 2.986 | 0.085 | -1.625 | 299 | 0.105 | -0.23271 | 0.14321 | -0.51455 | 0.04912 |
|  | Equal variances not assumed |  |  | -1.373 | 73.372 | 0.174 | -0.23271 | 0.16949 | -0.57047 | 0.10504 |
| R9 | Equal variances assumed | 0.078 | 0.781 | -0.316 | 299 | 0.752 | -0.02869 | 0.09072 | -0.20723 | 0.14985 |
|  | Equal variances not assumed |  |  | -0.29 | 78.853 | 0.772 | -0.02869 | 0.09882 | -0.2254 | 0.16802 |
