# Supplementary material for: Quantitative analysis of the labia minora morphology in 400 Chinese women: A new method for assessing the shape of the labia minora
Source: Front Surg. 2023 Jan 6;9:961247. doi: 10.3389/fsurg.2022.961247 (PMC9852508; doi:10.3389/fsurg.2022.961247)
Supplement: Supplementary file 3 [file Table3.docx]

**Supplementary file 3. Group statistics and independent samples t-tests for Group I & Group II.** Group I: Patients who underwent labiaplasty (either unilateral or bilateral). Group II: Patient who did not undergo labiaplasty.

| **Group I & Group II** | | | | | |
| --- | --- | --- | --- | --- | --- |
|  |  | N | Mean | Std. Deviation | Std. Error Mean |
| L1 | Group I | 223 | 1.1463 | 0.82372 | 0.05516 |
|  | Group II | 177 | 1.126 | 0.63389 | 0.04765 |
| L2 | Group I | 223 | 2.3263 | 1.28134 | 0.0858 |
|  | Group II | 177 | 2.2986 | 1.19106 | 0.08953 |
| L3 | Group I | 223 | 3.0196 | 1.26593 | 0.08477 |
|  | Group II | 177 | 3.0633 | 1.28885 | 0.09688 |
| L4 | Group I | 223 | 3.3433 | 1.13895 | 0.07627 |
|  | Group II | 177 | 3.3191 | 1.1462 | 0.08615 |
| L5 | Group I | 223 | 3.2494 | 1.13903 | 0.07627 |
|  | Group II | 177 | 3.1284 | 1.03715 | 0.07796 |
| L6 | Group I | 223 | 2.8406 | 1.00091 | 0.06703 |
|  | Group II | 177 | 2.7122 | 0.94094 | 0.07073 |
| L7 | Group I | 223 | 2.1134 | 1.05322 | 0.07053 |
|  | Group II | 177 | 1.9903 | 0.99257 | 0.07461 |
| L8 | Group I | 223 | 1.8463 | 0.98128 | 0.06571 |
|  | Group II | 177 | 1.7052 | 0.84389 | 0.06343 |
| L9 | Group I | 223 | 1.3279 | 0.68817 | 0.04608 |
|  | Group II | 177 | 1.297 | 0.66011 | 0.04962 |
| R1 | Group I | 223 | 0.9479 | 0.61031 | 0.04087 |
|  | Group II | 177 | 0.9321 | 0.54649 | 0.04108 |
| R2 | Group I | 223 | 2.1874 | 1.09062 | 0.07303 |
|  | Group II | 177 | 2.2943 | 1.15193 | 0.08658 |
| R3 | Group I | 223 | 3.034 | 1.15525 | 0.07736 |
|  | Group II | 177 | 3.0914 | 1.18403 | 0.089 |
| R4 | Group I | 223 | 3.0945 | 1.12877 | 0.07559 |
|  | Group II | 177 | 3.0571 | 1.09465 | 0.08228 |
| R5 | Group I | 223 | 2.8842 | 1.1585 | 0.07758 |
|  | Group II | 177 | 2.7917 | 1.07409 | 0.08073 |
| R6 | Group I | 223 | 2.3119 | 1.09432 | 0.07328 |
|  | Group II | 177 | 2.3163 | 1.06338 | 0.07993 |
| R7 | Group I | 223 | 1.7267 | 1.1062 | 0.07408 |
|  | Group II | 177 | 1.813 | 1.18189 | 0.08884 |
| R8 | Group I | 223 | 1.3962 | 0.92579 | 0.062 |
|  | Group II | 177 | 1.4331 | 1.01675 | 0.07642 |
| R9 | Group I | 223 | 0.9174 | 0.53002 | 0.03549 |
|  | Group II | 177 | 0.971 | 0.69327 | 0.05211 |

| **Group I & Group II** | | | | | | | | | | |
| --- | --- | --- | --- | --- | --- | --- | --- | --- | --- | --- |
|  |  | Levene's Test for Equality of Variances | | t-test for Equality of Means | | |  |  |  |  |
|  |  | F | Sig. | t | df | Sig. (2-tailed) | Mean Difference | Std.Error Difference | 95% Confidence interval of the Difference | |
|  |  |  |  |  |  |  |  |  | Lower | Upper |
| L1 | Equal variances assumed | 0.656 | 0.418 | 0.271 | 398 | 0.786 | 0.02038 | 0.07507 | -0.12721 | 0.16797 |
|  | Equal variances not assumed |  |  | 0.28 | 397.642 | 0.78 | 0.02038 | 0.07289 | -0.12291 | 0.16368 |
| L2 | Equal variances assumed | 0.755 | 0.385 | 0.221 | 398 | 0.825 | 0.02765 | 0.12505 | -0.2182 | 0.27349 |
|  | Equal variances not assumed |  |  | 0.223 | 388.177 | 0.824 | 0.02765 | 0.12401 | -0.21616 | 0.27145 |
| L3 | Equal variances assumed | 0.121 | 0.728 | -0.34 | 398 | 0.734 | -0.04372 | 0.12846 | -0.29627 | 0.20883 |
|  | Equal variances not assumed |  |  | -0.34 | 374.601 | 0.734 | -0.04372 | 0.12873 | -0.29684 | 0.20941 |
| L4 | Equal variances assumed | 0.091 | 0.764 | 0.21 | 398 | 0.834 | 0.02417 | 0.11498 | -0.20187 | 0.25021 |
|  | Equal variances not assumed |  |  | 0.21 | 376.591 | 0.834 | 0.02417 | 0.11506 | -0.20207 | 0.25042 |
| L5 | Equal variances assumed | 1.524 | 0.218 | 1.097 | 398 | 0.273 | 0.12098 | 0.11025 | -0.09576 | 0.33772 |
|  | Equal variances not assumed |  |  | 1.109 | 390.529 | 0.268 | 0.12098 | 0.10907 | -0.09345 | 0.33541 |
| L6 | Equal variances assumed | 1.576 | 0.21 | 1.308 | 398 | 0.191 | 0.12841 | 0.09814 | -0.06452 | 0.32133 |
|  | Equal variances not assumed |  |  | 1.318 | 386.77 | 0.188 | 0.12841 | 0.09744 | -0.06317 | 0.31998 |
| L7 | Equal variances assumed | 2.563 | 0.11 | 1.191 | 398 | 0.234 | 0.12309 | 0.10337 | -0.08013 | 0.32631 |
|  | Equal variances not assumed |  |  | 1.199 | 386.45 | 0.231 | 0.12309 | 0.10267 | -0.07876 | 0.32494 |
| L8 | Equal variances assumed | 3.877 | 0.05 | 1.518 | 398 | 0.13 | 0.14105 | 0.09292 | -0.04162 | 0.32373 |
|  | Equal variances not assumed |  |  | 1.544 | 395.418 | 0.123 | 0.14105 | 0.09133 | -0.0385 | 0.32061 |
| L9 | Equal variances assumed | 1.907 | 0.168 | 0.454 | 398 | 0.65 | 0.03092 | 0.06804 | -0.10285 | 0.16468 |
|  | Equal variances not assumed |  |  | 0.457 | 384.047 | 0.648 | 0.03092 | 0.06772 | -0.10222 | 0.16406 |
| R1 | Equal variances assumed | 0.356 | 0.551 | 0.269 | 398 | 0.788 | 0.01581 | 0.05868 | -0.09956 | 0.13118 |
|  | Equal variances not assumed |  |  | 0.273 | 392.215 | 0.785 | 0.01581 | 0.05794 | -0.09811 | 0.12973 |
| R2 | Equal variances assumed | 1.809 | 0.179 | -0.95 | 398 | 0.343 | -0.10688 | 0.11256 | -0.32817 | 0.11441 |
|  | Equal variances not assumed |  |  | -0.944 | 367.892 | 0.346 | -0.10688 | 0.11327 | -0.32962 | 0.11586 |
| R3 | Equal variances assumed | 1.63 | 0.202 | -0.488 | 398 | 0.626 | -0.05739 | 0.11759 | -0.28856 | 0.17378 |
|  | Equal variances not assumed |  |  | -0.487 | 373.429 | 0.627 | -0.05739 | 0.11792 | -0.28926 | 0.17448 |
| R4 | Equal variances assumed | 0.153 | 0.696 | 0.334 | 398 | 0.738 | 0.0375 | 0.11212 | -0.18293 | 0.25793 |
|  | Equal variances not assumed |  |  | 0.336 | 382.465 | 0.737 | 0.0375 | 0.11173 | -0.18218 | 0.25718 |
| R5 | Equal variances assumed | 0.943 | 0.332 | 0.819 | 398 | 0.413 | 0.09249 | 0.11294 | -0.12956 | 0.31453 |
|  | Equal variances not assumed |  |  | 0.826 | 388.49 | 0.409 | 0.09249 | 0.11197 | -0.12765 | 0.31262 |
| R6 | Equal variances assumed | 0.113 | 0.737 | -0.04 | 398 | 0.968 | -0.00438 | 0.1088 | -0.21827 | 0.20951 |
|  | Equal variances not assumed |  |  | -0.04 | 382.166 | 0.968 | -0.00438 | 0.10844 | -0.21759 | 0.20883 |
| R7 | Equal variances assumed | 0.487 | 0.486 | -0.752 | 398 | 0.452 | -0.08635 | 0.11479 | -0.31203 | 0.13932 |
|  | Equal variances not assumed |  |  | -0.747 | 365.68 | 0.456 | -0.08635 | 0.11567 | -0.31381 | 0.14111 |
| R8 | Equal variances assumed | 0.324 | 0.569 | -0.379 | 398 | 0.705 | -0.03692 | 0.09735 | -0.22831 | 0.15447 |
|  | Equal variances not assumed |  |  | -0.375 | 360.195 | 0.708 | -0.03692 | 0.09841 | -0.23044 | 0.15661 |
| R9 | Equal variances assumed | 1.835 | 0.176 | -0.877 | 398 | 0.381 | -0.05362 | 0.06117 | -0.17388 | 0.06664 |
|  | Equal variances not assumed |  |  | -0.85 | 322.205 | 0.396 | -0.05362 | 0.06305 | -0.17766 | 0.07042 |
