# Supplementary material for: Quantitative analysis of the labia minora morphology in 400 Chinese women: A new method for assessing the shape of the labia minora
Source: Front Surg. 2023 Jan 6;9:961247. doi: 10.3389/fsurg.2022.961247 (PMC9852508; doi:10.3389/fsurg.2022.961247)
Supplement: Supplementary file 4 [file Table4.docx]

**Supplementary file 4. Clinical characteristics of included patients.**

|  | | | | Surgical Information | | | | | | | |
| --- | --- | --- | --- | --- | --- | --- | --- | --- | --- | --- | --- |
| Serial Number | Date of surgery | Age | Number of births | left side Labiaplasty | Right side Labiaplasty | Labiaplasty of both sides | Clitoral hood plastic procedure | Vaginal tightening surgery | Labia majora augmentation | Hymen repair | Other procedures on the vulva area |
| 1 | 2021-01-04 | 27 | 0 |  |  | √ | √ |  |  |  |  |
| 2 | 2021-01-08 | 25 | 0 |  |  |  |  |  |  | √ |  |
| 3 | 2021-01-13 | 33 | 1 |  |  |  |  |  | √ |  |  |
| 4 | 2021-01-13 | 41 | 1 |  |  |  |  | √ |  |  |  |
| 5 | 2021-01-15 | 23 | 0 |  |  | √ | √ |  |  | √ |  |
| 6 | 2021-01-22 | 21 | 0 | √ |  |  |  |  |  |  |  |
| 7 | 2021-01-22 | 38 | 1 |  |  |  |  | √ |  |  |  |
| 8 | 2021-01-25 | 23 | 0 |  | √ |  | √ |  |  |  |  |
| 9 | 2021-01-26 | 24 | 0 |  |  |  |  |  |  |  |  |
| 10 | 2021-01-28 | 35 | 1 |  |  |  |  | √ |  |  |  |
| 11 | 2021-02-01 | 23 | 0 |  |  | √ | √ |  |  |  |  |
| 12 | 2021-02-05 | 35 | 0 |  |  |  |  |  |  |  |  |
| 13 | 2021-02-05 | 34 | 0 |  |  | √ | √ |  |  |  |  |
| 14 | 2021-02-07 | 33 | 1 |  |  |  |  | √ |  |  |  |
| 15 | 2021-02-07 | 33 | 1 |  |  |  |  | √ |  |  |  |
| 16 | 2021-02-08 | 33 | 0 |  |  | √ | √ |  |  |  |  |
| 17 | 2021-02-09 | 31 | 1 |  |  | √ | √ | √ | √ |  |  |
| 18 | 2021-02-19 | 36 | 1 |  |  |  |  | √ |  |  |  |
| 19 | 2021-02-22 | 25 | 0 |  |  |  |  | √ |  | √ |  |
| 20 | 2021-02-24 | 30 | 1 |  |  |  |  | √ |  |  |  |
| 21 | 2021-02-26 | 22 | 0 |  |  |  |  |  |  | √ |  |
| 22 | 2021-02-26 | 23 | 0 |  | √ |  | √ |  |  |  |  |
| 23 | 2021-02-26 | 28 | 0 |  |  | √ |  |  |  |  |  |
| 24 | 2021-03-02 | 29 | 0 | √ |  |  | √ |  |  |  |  |
| 25 | 2021-03-02 | 31 | 1 |  |  |  |  | √ |  |  |  |
| 26 | 2021-03-03 | 32 | 1 |  |  | √ | √ | √ | √ |  |  |
| 27 | 2021-03-05 | 31 | 2 |  |  |  |  | √ |  |  |  |
| 28 | 2021-03-05 | 30 | 0 |  |  | √ | √ |  |  |  |  |
| 29 | 2021-03-08 | 29 | 0 |  |  | √ |  |  |  |  |  |
| 30 | 2021-03-09 | 34 | 1 |  |  |  |  | √ |  |  |  |
| 31 | 2021-03-09 | 29 | 0 |  |  | √ | √ |  |  |  |  |
| 32 | 2021-03-09 | 28 | 0 |  |  | √ |  |  |  |  |  |
| 33 | 2021-03-09 | 40 | 2 |  |  |  |  | √ |  |  |  |
| 34 | 2021-03-09 | 34 | 1 |  | √ |  | √ | √ |  |  |  |
| 35 | 2021-03-09 | 28 | 1 | √ |  |  | √ |  | √ |  |  |
| 36 | 2021-03-09 | 33 | 0 | √ |  |  | √ |  |  |  |  |
| 37 | 2021-03-09 | 35 | 0 |  |  |  |  | √ |  |  |  |
| 38 | 2021-03-09 | 26 | 0 |  |  |  |  |  |  | √ |  |
| 39 | 2021-03-09 | 35 | 0 |  |  | √ | √ |  |  |  |  |
| 40 | 2021-03-16 | 36 | 1 |  |  |  |  | √ |  |  |  |
| 41 | 2021-03-16 | 36 | 0 |  |  |  |  |  |  |  |  |
| 42 | 2021-03-22 | 30 | 1 |  |  |  |  | √ |  |  |  |
| 43 | 2021-03-24 | 38 | 0 |  |  |  |  |  |  |  | √ |
| 44 | 2021-03-25 | 37 | 1 |  |  |  |  | √ |  |  |  |
| 45 | 2021-03-26 | 22 | 0 |  |  | √ | √ |  |  |  |  |
| 46 | 2021-03-30 | 19 | 0 |  |  | √ |  |  |  |  |  |
| 47 | 2021-03-30 | 40 | 3 |  |  |  |  | √ |  |  |  |
| 48 | 2021-04-01 | 22 | 0 |  |  | √ | √ |  |  |  |  |
| 49 | 2021-04-01 | 20 | 0 |  |  | √ |  |  |  |  |  |
| 50 | 2021-04-02 | 18 | 0 |  |  | √ | √ |  |  |  |  |
| 51 | 2021-04-02 | 28 | 1 |  |  | √ | √ |  | √ |  |  |
| 52 | 2021-04-02 | 20 | 0 |  |  | √ | √ |  |  |  |  |
| 53 | 2021-04-06 | 42 | 2 |  |  | √ | √ | √ |  |  |  |
| 54 | 2021-04-06 | 22 | 0 |  |  | √ | √ |  |  |  |  |
| 55 | 2021-04-06 | 20 | 0 | √ |  |  | √ |  |  | √ |  |
| 56 | 2021-04-06 | 35 | 0 |  |  |  |  |  |  |  | √ |
| 57 | 2021-04-08 | 21 | 0 |  |  |  |  |  |  | √ |  |
| 58 | 2021-04-13 | 43 | 1 |  |  |  |  | √ |  |  |  |
| 59 | 2021-04-13 | 34 | 0 |  |  | √ | √ |  |  |  |  |
| 60 | 2021-04-14 | 30 | 0 |  |  |  |  |  |  |  | √ |
| 61 | 2021-04-14 | 27 | 0 |  |  | √ | √ |  |  |  |  |
| 62 | 2021-04-14 | 44 | 1 |  |  |  |  | √ |  |  |  |
| 63 | 2021-04-16 | 39 | 0 |  |  | √ | √ |  |  |  |  |
| 64 | 2021-04-16 | 37 | 0 |  | √ |  | √ |  |  |  |  |
| 65 | 2021-04-19 | 21 | 0 |  |  | √ |  |  |  |  |  |
| 66 | 2021-04-19 | 22 | 0 |  |  | √ | √ |  |  |  |  |
| 67 | 2021-04-20 | 21 | 0 |  |  | √ |  |  |  |  |  |
| 68 | 2021-04-22 | 39 | 1 |  |  |  |  | √ |  |  |  |
| 69 | 2021-04-23 | 29 | 0 |  | √ |  | √ |  |  |  |  |
| 70 | 2021-04-26 | 25 | 0 |  |  |  |  | √ |  |  |  |
| 71 | 2021-04-26 | 23 | 0 |  |  |  |  |  |  | √ |  |
| 72 | 2021-04-27 | 32 | 0 |  |  | √ | √ |  |  |  |  |
| 73 | 2021-04-27 | 39 | 0 |  |  | √ |  |  |  |  |  |
| 74 | 2021-04-27 | 23 | 0 |  |  | √ |  |  |  |  |  |
| 75 | 2021-04-27 | 24 | 0 |  |  |  |  |  |  | √ |  |
| 76 | 2021-04-28 | 30 | 1 |  |  | √ | √ |  | √ |  |  |
| 77 | 2021-04-30 | 28 | 0 |  |  | √ | √ |  |  |  |  |
| 78 | 2021-05-07 | 36 | 1 |  |  | √ |  |  | √ |  |  |
| 79 | 2021-05-08 | 26 | 1 |  |  |  |  | √ |  |  |  |
| 80 | 2021-05-11 | 21 | 0 |  |  |  |  |  |  |  |  |
| 81 | 2021-05-11 | 22 | 0 |  |  | √ | √ |  |  |  |  |
| 82 | 2021-05-11 | 29 | 0 |  |  |  |  |  |  |  | √ |
| 83 | 2021-05-12 | 23 | 0 |  |  | √ | √ |  |  |  |  |
| 84 | 2021-05-12 | 32 | 2 |  |  |  |  | √ |  |  |  |
| 85 | 2021-05-13 | 23 | 0 |  |  | √ | √ |  |  |  |  |
| 86 | 2021-05-14 | 24 | 0 |  |  | √ | √ |  |  |  |  |
| 87 | 2021-05-14 | 27 | 1 |  |  |  |  | √ |  |  |  |
| 88 | 2021-05-17 | 29 | 0 |  |  |  |  |  |  |  | √ |
| 89 | 2021-05-17 | 30 | 1 |  |  |  | √ |  |  |  |  |
| 90 | 2021-05-18 | 29 | 0 |  |  | √ | √ |  |  |  |  |
| 91 | 2021-05-18 | 31 | 1 |  |  | √ | √ |  |  |  |  |
| 92 | 2021-05-18 | 43 | 0 |  |  | √ | √ |  |  |  |  |
| 93 | 2021-05-18 | 42 | 0 |  |  | √ | √ |  |  |  |  |
| 94 | 2021-05-19 | 28 | 0 |  |  |  |  |  |  | √ |  |
| 95 | 2021-05-19 | 40 | 0 |  |  | √ | √ |  |  |  |  |
| 96 | 2021-05-19 | 41 | 0 |  |  | √ | √ |  |  |  |  |
| 97 | 2021-05-20 | 44 | 0 |  |  | √ | √ |  |  |  |  |
| 98 | 2021-05-24 | 30 | 2 |  |  |  |  | √ |  |  |  |
| 99 | 2021-05-25 | 29 | 1 |  |  | √ | √ | √ |  |  |  |
| 100 | 2021-05-26 | 51 | 2 |  |  | √ | √ |  |  |  |  |
| 101 | 2021-05-27 | 30 | 0 |  |  |  |  |  | √ |  |  |
| 102 | 2021-05-27 | 29 | 0 |  |  |  |  |  |  | √ |  |
| 103 | 2021-05-28 | 23 | 0 |  |  | √ | √ |  |  |  |  |
| 104 | 2021-05-31 | 49 | 3 |  |  | √ |  |  |  |  |  |
| 105 | 2021-06-02 | 27 | 0 |  |  |  |  |  |  | √ |  |
| 106 | 2021-06-02 | 21 | 0 |  |  | √ | √ |  |  |  |  |
| 107 | 2021-06-04 | 35 | 0 | √ |  |  | √ |  |  |  |  |
| 108 | 2021-06-04 | 20 | 0 |  |  |  |  |  |  |  | √ |
| 109 | 2021-06-04 | 29 | 0 |  |  | √ | √ |  |  |  |  |
| 110 | 2021-06-07 | 28 | 0 |  |  | √ | √ |  |  |  |  |
| 111 | 2021-06-07 | 22 | 0 |  |  |  |  |  |  |  | √ |
| 112 | 2021-06-08 | 37 | 2 |  |  |  |  | √ |  |  |  |
| 113 | 2021-06-08 | 28 | 0 |  |  |  |  |  |  | √ |  |
| 114 | 2021-06-10 | 39 | 2 |  |  |  |  | √ |  |  |  |
| 115 | 2021-06-10 | 41 | 1 |  |  |  |  | √ |  |  |  |
| 116 | 2021-06-11 | 19 | 0 |  |  |  |  |  |  | √ |  |
| 117 | 2021-06-15 | 23 | 0 |  |  | √ | √ |  |  |  |  |
| 118 | 2021-06-15 | 20 | 0 |  |  |  |  |  |  |  |  |
| 119 | 2021-06-15 | 60 | 0 |  |  |  |  |  |  |  | √ |
| 120 | 2021-06-18 | 40 | 0 |  |  |  |  | √ |  |  |  |
| 121 | 2021-06-18 | 22 | 0 |  |  | √ |  |  |  |  |  |
| 122 | 2021-06-21 | 26 | 0 |  | √ |  | √ |  |  |  |  |
| 123 | 2021-06-22 | 26 | 0 |  | √ |  |  |  |  |  | √ |
| 124 | 2021-06-22 | 32 | 1 |  |  | √ | √ |  |  |  |  |
| 125 | 2021-06-22 | 26 | 0 |  |  |  |  |  |  | √ |  |
| 126 | 2021-06-22 | 34 | 0 | √ |  |  | √ |  | √ |  |  |
| 127 | 2021-06-22 | 46 | 2 |  |  |  |  | √ |  |  |  |
| 128 | 2021-06-22 | 38 | 1 |  |  |  |  |  |  |  | √ |
| 129 | 2021-06-22 | 28 | 1 |  |  |  |  |  |  | √ |  |
| 130 | 2021-06-25 | 47 | 1 |  |  |  |  | √ |  |  |  |
| 131 | 2021-06-25 | 28 | 0 |  |  | √ | √ |  |  |  |  |
| 132 | 2021-06-29 | 45 | 1 |  |  | √ |  | √ |  |  |  |
| 133 | 2021-06-29 | 24 | 0 |  |  |  |  |  |  | √ |  |
| 134 | 2021-06-30 | 21 | 0 |  |  |  |  |  | √ |  |  |
| 135 | 2021-06-30 | 30 | 0 |  |  | √ | √ |  |  |  |  |
| 136 | 2021-07-01 | 26 | 0 |  |  |  |  |  |  | √ |  |
| 137 | 2021-07-02 | 22 | 0 |  |  | √ | √ |  |  |  |  |
| 138 | 2021-07-05 | 28 | 0 |  |  | √ | √ |  |  |  |  |
| 139 | 2021-07-05 | 21 | 0 |  |  | √ |  |  |  |  |  |
| 140 | 2021-07-05 | 44 | 2 |  |  |  |  | √ |  |  |  |
| 141 | 2021-07-06 | 26 | 0 |  | √ |  | √ |  |  |  |  |
| 142 | 2021-07-06 | 22 | 0 |  |  |  |  |  |  | √ |  |
| 143 | 2021-07-06 | 39 | 1 |  |  |  |  | √ |  |  |  |
| 144 | 2021-07-06 | 40 | 0 |  | √ |  | √ |  | √ |  |  |
| 145 | 2021-07-07 | 39 | 0 | √ |  |  | √ | √ |  |  |  |
| 146 | 2021-07-08 | 36 | 1 |  |  | √ | √ |  |  |  |  |
| 147 | 2021-07-08 | 23 | 0 |  |  | √ | √ |  |  |  |  |
| 148 | 2021-07-08 | 37 | 1 |  |  |  |  | √ |  |  |  |
| 149 | 2021-07-08 | 22 | 0 |  |  | √ | √ |  |  |  |  |
| 150 | 2021-07-10 | 23 | 0 |  |  | √ | √ |  |  |  |  |
| 151 | 2021-07-10 | 36 | 2 |  |  |  |  | √ |  |  |  |
| 152 | 2021-07-10 | 28 | 0 |  |  | √ | √ |  |  |  |  |
| 153 | 2021-07-10 | 29 | 0 |  |  | √ | √ |  |  |  |  |
| 154 | 2021-07-10 | 33 | 0 | √ |  |  | √ |  |  |  |  |
| 155 | 2021-07-12 | 34 | 0 |  |  | √ | √ |  |  |  |  |
| 156 | 2021-07-12 | 35 | 2 |  |  |  |  | √ |  |  |  |
| 157 | 2021-07-12 | 23 | 0 |  |  | √ | √ |  |  |  |  |
| 158 | 2021-07-12 | 32 | 1 |  |  |  |  | √ |  |  |  |
| 159 | 2021-07-12 | 33 | 1 |  |  |  |  | √ |  |  |  |
| 160 | 2021-07-14 | 30 | 0 |  |  |  |  |  | √ |  |  |
| 161 | 2021-07-14 | 28 | 0 |  |  | √ | √ |  |  |  |  |
| 162 | 2021-07-14 | 21 | 0 |  |  | √ | √ |  |  |  |  |
| 163 | 2021-07-14 | 29 | 2 |  |  |  |  | √ |  |  |  |
| 164 | 2021-07-19 | 23 | 0 |  | √ |  | √ |  |  |  |  |
| 165 | 2021-07-19 | 32 | 1 |  |  |  |  | √ |  |  |  |
| 166 | 2021-07-19 | 31 | 1 |  |  |  |  | √ |  |  |  |
| 167 | 2021-07-19 | 21 | 0 |  |  | √ | √ |  |  |  |  |
| 168 | 2021-07-21 | 40 | 2 |  |  | √ |  |  |  |  |  |
| 169 | 2021-07-23 | 45 | 3 |  |  | √ | √ |  |  |  |  |
| 170 | 2021-07-23 | 23 | 0 |  | √ |  | √ |  |  |  |  |
| 171 | 2021-07-26 | 24 | 0 |  |  | √ |  |  |  |  |  |
| 172 | 2021-07-26 | 37 | 1 |  |  | √ | √ | √ | √ |  |  |
| 173 | 2021-07-26 | 23 | 0 |  |  |  |  |  |  | √ |  |
| 174 | 2021-07-26 | 28 | 0 |  |  | √ | √ |  | √ |  |  |
| 175 | 2021-07-27 | 23 | 0 |  |  |  |  |  |  |  | √ |
| 176 | 2021-07-27 | 30 | 1 |  |  | √ | √ |  |  |  |  |
| 177 | 2021-07-27 | 31 | 1 |  |  | √ | √ |  |  |  |  |
| 178 | 2021-07-27 | 36 | 0 |  |  | √ | √ |  |  |  |  |
| 179 | 2021-07-28 | 37 | 1 |  |  | √ | √ |  |  |  |  |
| 180 | 2021-07-28 | 36 | 1 |  |  |  |  | √ |  |  |  |
| 181 | 2021-07-28 | 22 | 0 |  |  | √ | √ |  |  | √ |  |
| 182 | 2021-07-28 | 35 | 0 |  |  | √ | √ |  | √ |  |  |
| 183 | 2021-07-28 | 21 | 0 |  |  |  |  |  |  | √ |  |
| 184 | 2021-07-29 | 19 | 0 |  |  |  |  |  |  | √ |  |
| 185 | 2021-07-29 | 30 | 0 |  |  | √ | √ |  |  |  |  |
| 186 | 2021-08-02 | 31 | 0 |  |  | √ | √ |  |  |  |  |
| 187 | 2021-08-03 | 36 | 2 |  |  |  |  | √ |  |  |  |
| 188 | 2021-08-03 | 37 | 1 |  |  |  |  | √ |  |  |  |
| 189 | 2021-08-03 | 38 | 2 |  |  |  |  | √ |  |  |  |
| 190 | 2021-08-03 | 38 | 1 | √ |  |  | √ | √ | √ |  |  |
| 191 | 2021-08-03 | 20 | 0 |  |  |  |  |  |  | √ |  |
| 192 | 2021-08-03 | 28 | 0 |  |  | √ | √ |  |  |  |  |
| 193 | 2021-08-03 | 29 | 0 |  |  |  |  |  |  | √ |  |
| 194 | 2021-08-03 | 47 | 2 |  |  |  |  | √ |  |  |  |
| 195 | 2021-08-04 | 27 | 0 |  |  | √ | √ |  | √ |  |  |
| 196 | 2021-08-05 | 26 | 0 |  |  | √ | √ |  |  |  |  |
| 197 | 2021-08-05 | 41 | 1 |  |  |  |  | √ |  |  |  |
| 198 | 2021-08-05 | 23 | 0 |  |  | √ |  |  |  |  |  |
| 199 | 2021-08-05 | 40 | 1 |  |  |  |  | √ |  |  |  |
| 200 | 2021-08-06 | 40 | 1 |  |  |  |  | √ |  |  |  |
| 201 | 2021-08-06 | 22 | 0 | √ |  |  | √ |  |  |  |  |
| 202 | 2021-08-06 | 35 | 0 |  |  |  |  |  |  |  | √ |
| 203 | 2021-08-10 | 34 | 0 |  |  |  |  |  |  |  |  |
| 204 | 2021-08-10 | 28 | 0 |  |  |  |  |  |  | √ |  |
| 205 | 2021-08-10 | 25 | 1 |  |  |  |  |  |  | √ |  |
| 206 | 2021-08-10 | 26 | 1 |  |  |  |  |  |  | √ |  |
| 207 | 2021-08-10 | 23 | 0 |  |  | √ | √ |  |  |  |  |
| 208 | 2021-08-11 | 29 | 0 |  |  | √ | √ |  |  |  |  |
| 209 | 2021-08-11 | 30 | 0 |  |  | √ | √ |  |  |  |  |
| 210 | 2021-08-11 | 24 | 1 |  |  | √ |  |  |  | √ |  |
| 211 | 2021-08-16 | 22 | 0 |  |  |  |  |  |  | √ |  |
| 212 | 2021-08-16 | 23 | 0 |  |  |  |  |  |  | √ |  |
| 213 | 2021-08-16 | 33 | 1 |  | √ |  | √ |  |  |  |  |
| 214 | 2021-08-17 | 25 | 0 |  |  |  |  |  |  |  | √ |
| 215 | 2021-08-20 | 27 | 0 |  |  |  |  |  |  | √ |  |
| 216 | 2021-08-20 | 22 | 0 |  |  | √ | √ |  |  |  |  |
| 217 | 2021-08-23 | 21 | 0 |  |  | √ | √ |  |  |  |  |
| 218 | 2021-08-23 | 31 | 2 |  |  | √ |  | √ | √ |  |  |
| 219 | 2021-08-24 | 20 | 0 |  |  | √ | √ |  |  |  |  |
| 220 | 2021-08-27 | 20 | 0 | √ |  |  | √ |  |  |  |  |
| 221 | 2021-08-30 | 28 | 2 |  |  |  |  | √ |  | √ |  |
| 222 | 2021-08-30 | 35 | 0 |  |  |  | √ |  |  |  |  |
| 223 | 2021-08-30 | 29 | 2 |  |  | √ | √ | √ |  |  |  |
| 224 | 2021-08-31 | 36 | 0 |  |  |  |  |  |  |  | √ |
| 225 | 2021-08-31 | 27 | 2 |  |  |  |  | √ |  |  |  |
| 226 | 2021-09-02 | 28 | 2 |  |  |  |  | √ |  | √ |  |
| 227 | 2021-09-07 | 30 | 1 |  |  | √ | √ |  | √ |  |  |
| 228 | 2021-09-07 | 31 | 0 |  |  | √ | √ |  |  |  |  |
| 229 | 2021-09-07 | 32 | 2 |  |  |  |  | √ |  | √ |  |
| 230 | 2021-09-07 | 35 | 0 |  |  | √ | √ |  | √ |  |  |
| 231 | 2021-09-08 | 28 | 0 |  |  | √ | √ |  |  |  |  |
| 232 | 2021-09-09 | 26 | 0 |  |  | √ | √ |  |  |  |  |
| 233 | 2021-09-13 | 20 | 2 |  |  | √ | √ |  |  | √ |  |
| 234 | 2021-09-13 | 25 | 0 |  |  | √ | √ |  | √ |  |  |
| 235 | 2021-09-13 | 26 | 0 |  |  | √ | √ |  |  |  |  |
| 236 | 2021-09-13 | 28 | 0 |  | √ |  |  | √ |  |  |  |
| 237 | 2021-09-14 | 37 | 0 |  |  | √ | √ |  | √ |  |  |
| 238 | 2021-09-14 | 30 | 0 |  |  |  |  | √ |  |  |  |
| 239 | 2021-09-15 | 27 | 1 |  |  | √ | √ | √ |  |  |  |
| 240 | 2021-09-16 | 43 | 1 |  |  | √ | √ |  |  |  |  |
| 241 | 2021-09-18 | 44 | 2 |  |  | √ | √ |  |  |  |  |
| 242 | 2021-09-18 | 20 | 0 |  |  | √ | √ |  |  |  |  |
| 243 | 2021-09-18 | 45 | 2 |  |  | √ | √ |  |  |  |  |
| 244 | 2021-09-18 | 29 | 0 |  |  |  |  |  | √ |  |  |
| 245 | 2021-09-23 | 29 | 0 |  | √ |  | √ |  |  |  |  |
| 246 | 2021-09-24 | 28 | 0 |  |  |  |  | √ |  |  |  |
| 247 | 2021-09-24 | 29 | 0 |  |  |  |  | √ |  |  |  |
| 248 | 2021-09-24 | 25 | 0 |  |  |  |  |  |  |  | √ |
| 249 | 2021-09-26 | 23 | 0 |  |  | √ | √ |  |  |  |  |
| 250 | 2021-09-27 | 32 | 0 |  | √ |  | √ |  |  |  |  |
| 251 | 2021-09-27 | 34 | 2 |  |  | √ | √ | √ |  |  |  |
| 252 | 2021-09-27 | 35 | 2 |  |  |  |  | √ |  |  |  |
| 253 | 2021-09-28 | 33 | 0 | √ |  |  | √ |  |  |  |  |
| 254 | 2021-09-29 | 23 | 0 |  |  |  |  |  |  | √ |  |
| 255 | 2021-09-30 | 33 | 1 |  |  |  |  |  |  |  | √ |
| 256 | 2021-09-30 | 24 | 0 |  |  |  |  |  |  | √ |  |
| 257 | 2021-10-09 | 34 | 1 | √ |  |  | √ |  |  |  |  |
| 258 | 2021-10-09 | 31 | 1 |  |  | √ | √ |  |  |  |  |
| 259 | 2021-10-09 | 30 | 0 |  |  |  |  |  |  |  | √ |
| 260 | 2021-10-11 | 35 | 1 |  |  | √ | √ |  |  |  |  |
| 261 | 2021-10-11 | 24 | 0 |  |  | √ | √ |  |  |  |  |
| 262 | 2021-10-12 | 26 | 0 |  |  | √ |  |  |  |  |  |
| 263 | 2021-10-13 | 28 | 0 |  |  |  |  |  | √ |  |  |
| 264 | 2021-10-14 | 43 | 3 |  |  |  |  | √ |  |  |  |
| 265 | 2021-10-14 | 37 | 0 |  |  | √ | √ |  |  |  |  |
| 266 | 2021-10-14 | 38 | 0 |  |  | √ | √ |  |  |  |  |
| 267 | 2021-10-14 | 40 | 0 |  |  | √ |  |  |  |  |  |
| 268 | 2021-10-15 | 44 | 0 |  |  |  |  | √ |  |  |  |
| 269 | 2021-10-18 | 43 | 0 |  |  |  |  | √ | √ |  |  |
| 270 | 2021-10-18 | 32 | 1 |  |  | √ | √ |  |  |  |  |
| 271 | 2021-10-18 | 26 | 0 |  |  |  |  |  |  | √ |  |
| 272 | 2021-10-18 | 31 | 0 |  |  | √ | √ |  |  |  |  |
| 273 | 2021-10-19 | 33 | 0 |  |  | √ | √ |  |  |  |  |
| 274 | 2021-10-19 | 25 | 0 |  | √ |  | √ |  |  |  |  |
| 275 | 2021-10-19 | 23 | 0 |  |  |  |  |  |  | √ |  |
| 276 | 2021-10-20 | 24 | 0 |  |  |  |  |  |  | √ |  |
| 277 | 2021-10-20 | 29 | 0 |  |  | √ | √ |  |  |  |  |
| 278 | 2021-10-21 | 42 | 2 |  |  | √ | √ | √ | √ |  |  |
| 279 | 2021-10-21 | 39 | 1 | √ |  |  | √ | √ |  |  |  |
| 280 | 2021-10-21 | 26 | 0 |  | √ |  | √ |  |  |  |  |
| 281 | 2021-10-25 | 38 | 0 |  |  | √ |  | √ |  |  |  |
| 282 | 2021-10-25 | 22 | 0 |  | √ |  | √ |  |  |  |  |
| 283 | 2021-10-25 | 31 | 0 |  |  | √ | √ |  |  |  |  |
| 284 | 2021-10-25 | 32 | 0 |  |  | √ | √ |  | √ |  |  |
| 285 | 2021-10-28 | 25 | 0 |  |  |  |  |  |  | √ |  |
| 286 | 2021-11-01 | 37 | 2 |  |  |  |  | √ |  |  |  |
| 287 | 2021-11-01 | 38 | 2 |  |  |  |  | √ |  |  |  |
| 288 | 2021-11-01 | 25 | 0 |  | √ |  | √ |  |  |  |  |
| 289 | 2021-11-01 | 22 | 0 |  |  |  |  |  |  | √ |  |
| 290 | 2021-11-01 | 40 | 2 |  |  |  |  | √ |  |  |  |
| 291 | 2021-11-02 | 24 | 0 |  |  |  |  |  |  | √ |  |
| 292 | 2021-11-02 | 39 | 2 |  |  |  |  | √ |  |  |  |
| 293 | 2021-11-04 | 40 | 0 | √ |  |  | √ | √ |  |  |  |
| 294 | 2021-11-08 | 39 | 2 |  |  |  |  | √ |  |  |  |
| 295 | 2021-11-08 | 22 | 0 |  |  | √ | √ |  | √ |  |  |
| 296 | 2021-11-08 | 23 | 0 |  |  |  |  |  |  |  |  |
| 297 | 2021-11-08 | 24 | 0 |  |  | √ |  |  |  |  |  |
| 298 | 2021-11-09 | 25 | 0 |  |  | √ | √ |  |  |  |  |
| 299 | 2021-11-12 | 20 | 0 |  |  |  |  |  |  | √ |  |
| 300 | 2021-11-16 | 43 | 2 |  |  |  |  | √ |  |  |  |
| 301 | 2021-11-16 | 24 | 0 |  | √ |  | √ |  |  |  |  |
| 302 | 2021-11-17 | 42 | 2 |  |  |  |  | √ |  |  |  |
| 303 | 2021-11-17 | 35 | 1 |  |  | √ | √ | √ |  | √ |  |
| 304 | 2021-11-18 | 32 | 0 |  |  | √ | √ |  |  |  |  |
| 305 | 2021-11-18 | 24 | 0 |  |  |  |  | √ |  | √ |  |
| 306 | 2021-11-23 | 26 | 0 |  |  |  |  | √ |  |  |  |
| 307 | 2021-11-23 | 33 | 0 |  |  | √ | √ | √ |  |  |  |
| 308 | 2021-11-23 | 33 | 0 |  |  | √ | √ |  |  |  |  |
| 309 | 2021-11-25 | 34 | 2 | √ |  |  |  | √ |  |  |  |
| 310 | 2021-11-25 | 18 | 0 |  |  |  |  | √ |  | √ |  |
| 311 | 2021-11-25 | 24 | 0 |  |  |  |  |  |  | √ |  |
| 312 | 2021-11-25 | 36 | 0 |  |  | √ | √ |  |  |  |  |
| 313 | 2021-11-25 | 22 | 0 |  |  |  |  |  |  | √ |  |
| 314 | 2021-11-29 | 70 | 1 |  |  |  |  |  |  |  | √ |
| 315 | 2021-11-29 | 38 | 2 |  |  |  |  | √ |  |  |  |
| 316 | 2021-11-29 | 36 | 1 |  |  |  |  | √ |  |  |  |
| 317 | 2021-11-30 | 33 | 2 |  | √ |  |  |  |  |  |  |
| 318 | 2021-12-02 | 36 | 1 |  |  |  |  | √ |  |  |  |
| 319 | 2021-12-06 | 34 | 1 |  |  |  |  | √ |  |  |  |
| 320 | 2021-12-06 | 36 | 2 |  |  |  |  |  |  |  | √ |
| 321 | 2021-12-07 | 36 | 0 |  |  | √ | √ | √ |  |  |  |
| 322 | 2021-12-08 | 40 | 2 |  |  | √ | √ |  |  |  |  |
| 323 | 2021-12-09 | 34 | 0 |  |  |  |  | √ |  |  |  |
| 324 | 2021-12-13 | 38 | 0 |  |  |  |  | √ |  |  |  |
| 325 | 2021-12-13 | 43 | 2 |  |  | √ | √ |  |  |  |  |
| 326 | 2021-12-13 | 39 | 1 |  |  | √ | √ |  |  |  |  |
| 327 | 2021-12-13 | 38 | 1 |  |  |  |  |  |  |  |  |
| 328 | 2021-12-13 | 26 | 1 |  |  |  |  |  |  | √ |  |
| 329 | 2021-12-16 | 30 | 1 |  |  |  |  | √ |  |  |  |
| 330 | 2021-12-17 | 34 | 2 |  |  |  |  | √ |  |  |  |
| 331 | 2021-12-17 | 22 | 0 |  |  | √ | √ |  |  |  |  |
| 332 | 2021-12-17 | 25 | 0 |  |  |  |  |  |  | √ |  |
| 333 | 2021-12-20 | 35 | 1 |  |  |  |  | √ |  |  |  |
| 334 | 2021-12-21 | 22 | 1 |  |  |  |  |  |  |  |  |
| 335 | 2021-12-22 | 40 | 1 |  |  |  |  | √ |  |  |  |
| 336 | 2021-12-27 | 41 | 1 |  |  | √ | √ | √ | √ |  |  |
| 337 | 2021-12-27 | 23 | 0 |  |  | √ |  |  |  |  |  |
| 338 | 2021-12-27 | 28 | 1 |  |  | √ |  |  |  |  |  |
| 339 | 2021-12-30 | 33 | 0 |  |  |  |  | √ |  |  |  |
| 340 | 2021-12-31 | 34 | 0 |  |  | √ | √ |  |  |  |  |
| 341 | 2022-01-04 | 31 | 1 |  |  | √ | √ |  |  |  |  |
| 342 | 2022-01-04 | 39 | 2 |  | √ |  |  | √ |  |  |  |
| 343 | 2022-01-06 | 30 | 0 |  | √ |  |  |  |  |  |  |
| 344 | 2022-01-06 | 49 | 2 |  |  |  |  | √ |  |  |  |
| 345 | 2022-01-06 | 23 | 1 |  |  | √ | √ |  |  |  |  |
| 346 | 2022-01-06 | 24 | 1 |  |  | √ | √ |  |  |  |  |
| 347 | 2022-01-06 | 52 | 2 |  | √ |  |  | √ |  |  |  |
| 348 | 2022-01-10 | 24 | 0 |  | √ |  |  |  |  |  |  |
| 349 | 2022-01-10 | 23 | 0 |  |  |  |  |  |  | √ |  |
| 350 | 2022-01-11 | 23 | 0 | √ |  |  |  |  |  | √ |  |
| 351 | 2022-01-11 | 51 | 2 |  |  |  |  | √ |  |  |  |
| 352 | 2022-01-11 | 24 | 0 |  |  | √ | √ |  |  |  |  |
| 353 | 2022-01-13 | 24 | 0 |  |  |  |  |  |  | √ |  |
| 354 | 2022-01-14 | 22 | 0 |  |  | √ | √ |  |  |  |  |
| 355 | 2022-01-14 | 30 | 1 |  |  | √ | √ |  |  |  |  |
| 356 | 2022-01-17 | 35 | 1 |  |  |  |  | √ |  |  |  |
| 357 | 2022-01-17 | 30 | 0 |  |  | √ | √ |  |  |  |  |
| 358 | 2022-01-17 | 23 | 0 |  |  | √ | √ |  |  |  |  |
| 359 | 2022-01-17 | 24 | 0 |  | √ |  |  |  |  |  |  |
| 360 | 2022-01-17 | 36 | 2 |  |  |  |  | √ |  |  |  |
| 361 | 2022-01-18 | 22 | 0 |  | √ |  | √ |  |  | √ |  |
| 362 | 2022-01-18 | 25 | 0 | √ |  |  | √ |  |  |  |  |
| 363 | 2022-01-18 | 26 | 1 |  |  | √ | √ |  |  |  |  |
| 364 | 2022-01-20 | 24 | 1 |  |  | √ | √ |  |  |  |  |
| 365 | 2022-01-20 | 23 | 0 |  | √ |  | √ |  |  |  |  |
| 366 | 2022-01-20 | 31 | 1 |  |  |  |  | √ |  |  |  |
| 367 | 2022-01-21 | 38 | 2 |  |  |  |  |  |  |  |  |
| 368 | 2022-01-21 | 30 | 1 |  |  | √ | √ |  |  |  |  |
| 369 | 2022-01-21 | 29 | 1 |  |  |  |  | √ |  |  |  |
| 370 | 2022-01-25 | 25 | 0 |  |  | √ |  |  |  |  |  |
| 371 | 2022-01-27 | 26 | 1 |  |  | √ | √ |  |  |  |  |
| 372 | 2022-02-07 | 35 | 2 |  |  |  |  | √ |  |  |  |
| 373 | 2022-02-08 | 21 | 0 |  |  |  |  |  |  | √ |  |
| 374 | 2022-02-09 | 21 | 0 |  |  |  |  |  |  | √ |  |
| 375 | 2022-02-10 | 23 | 1 | √ |  |  | √ |  |  |  |  |
| 376 | 2022-02-11 | 28 | 1 |  |  |  |  | √ |  |  | √ |
| 377 | 2022-02-11 | 35 | 2 |  |  | √ | √ |  |  |  |  |
| 378 | 2022-02-14 | 29 | 2 |  |  |  |  | √ |  |  |  |
| 379 | 2022-02-14 | 30 | 0 |  |  |  |  | √ |  |  |  |
| 380 | 2022-02-14 | 29 | 1 | √ |  |  | √ |  |  |  |  |
| 381 | 2022-02-14 | 30 | 0 |  |  |  |  | √ |  |  |  |
| 382 | 2022-02-15 | 28 | 0 |  |  | √ | √ |  |  |  |  |
| 383 | 2022-02-15 | 33 | 1 |  |  |  |  | √ |  |  |  |
| 384 | 2022-02-16 | 21 | 0 |  |  | √ |  |  |  |  |  |
| 385 | 2022-02-16 | 25 | 0 |  |  |  |  |  |  | √ |  |
| 386 | 2022-02-17 | 36 | 0 |  |  | √ |  | √ |  |  |  |
| 387 | 2022-02-17 | 29 | 1 |  |  | √ | √ |  |  |  |  |
| 388 | 2022-02-17 | 30 | 1 |  |  | √ | √ |  |  |  |  |
| 389 | 2022-02-17 | 26 | 0 |  |  |  |  |  |  | √ |  |
| 390 | 2022-02-18 | 26 | 0 |  |  | √ | √ |  |  |  |  |
| 391 | 2022-02-18 | 42 | 2 |  |  |  |  | √ |  |  |  |
| 392 | 2022-02-18 | 40 | 1 |  |  | √ | √ | √ |  |  |  |
| 393 | 2022-02-18 | 37 | 1 |  |  | √ | √ | √ |  |  |  |
| 394 | 2022-02-18 | 26 | 0 |  |  | √ | √ |  |  |  |  |
| 395 | 2022-02-21 | 24 | 0 |  |  | √ | √ |  |  |  |  |
| 396 | 2022-02-21 | 33 | 1 |  |  | √ | √ |  |  |  |  |
| 397 | 2022-02-22 | 26 | 0 |  |  | √ | √ |  |  |  |  |
| 398 | 2022-02-24 | 34 | 0 |  |  |  |  |  |  |  | √ |
| 399 | 2022-02-25 | 36 | 1 |  |  |  |  |  |  |  | √ |
| 400 | 2022-02-25 | 52 | 2 |  |  | √ | √ |  |  |  |  |
